# Outcome measurement for gender-affirming care in Canada: a systematic review

**DOI:** 10.1136/bmjopen-2024-091135

**Published:** 2025-03-12

**Authors:** Liam Jackman, Cynthia Chan, Micon Garvilles, Rakhshan Kamran

**Affiliations:** 1 University of Toronto Institute of Health Policy Management and Evaluation, Toronto, Ontario, Canada; 2 Department of Family and Community Medicine, University of Toronto, Toronto, Ontario, Canada; 3 Department of Orthopaedics Rheumatology and Musculoskeletal Sciences, University of Oxford Nuffield, Oxford, UK

**Keywords:** Clinical Decision-Making, Health Equity, Health Services, Quality in healthcare, Health Services for Transgender Persons

## Abstract

**Abstract:**

**Introduction:**

Gender-affirming care (GAC) includes interventions aimed at supporting an individual’s gender identity. Canada is experiencing an increase in referrals for GAC, higher than any other health service; therefore, there is a need for a systematic approach to health outcome measurement to effectively evaluate care. This review aims to analyse health outcome measurement in Canadian GAC, focusing on what is measured, how it is measured and associated barriers and enablers.

**Methods:**

A comprehensive search was conducted in MEDLINE, Embase, PsycINFO, Scopus and CINAHL, up to 26 December 2023. Inclusion criteria were original articles involving transgender or gender-diverse (TGD) patients receiving gender-affirming care in Canada.

**Results:**

A total of 4649 articles were identified with 64 included, representing 6561 TGD patients. Most studies were conducted in Ontario (52%), British Columbia (19%) and Quebec (11%). The most common forms of GAC provided were hormonal (36%) and surgical (27%). Barriers to outcome measurement include that most studies (61%) did not use patient-reported outcome measures (PROMs). When PROMs were used, most did not capture gender-related constructs (eg, gender dysphoria). Barriers to accessing care included stigma, discrimination, lack of clinician knowledge, geographic, socioeconomic and institutional barriers.

**Conclusion:**

This review reveals gaps in outcome measurement for GAC, particularly underutilisation of PROMs and inconsistent outcome measurement and reporting. There is a need to systematically implement PROMs, including those measuring gender-related constructs, to promote patient-centred care. This review provides evidence-based recommendations for improving health outcomes for TGD individuals in Canada.

**STRENGTHS AND LIMITATIONS OF THIS STUDY**

- This study provides a comprehensive analysis of outcome measurement (ie, performance indicators, physiological parameters, provider-reported outcomes and patient-reported outcomes) for gender-affirming care in Canada, barriers to care, and evidence-based recommendations for improvements in health outcome measurement for this clinical area following established guidelines for systematic reviews.
- This study was undertaken in partnership with patients and members of the public to ensure the meaningfulness of study results.
- A comprehensive and evidence-based search strategy was used to ensure the capture of relevant articles.
- This study focuses on Canadian gender-affirming care, and there might be limited generalisability of results to other countries, which may have different health systems.

## Introduction

 Gender-affirming care (GAC) includes social, psychological, medical and surgical interventions provided to affirm one’s gender and alleviate gender dysphoria.^1 2^ Canada’s national census identified over 100 000 transgender or gender-diverse (TGD) individuals with a higher proportion (1 in 150) among Canadians aged 15–34 and a lower proportion (1 in 550) among Canadians over the age of 35.^3^ Canada has seen a 10-fold increase in referrals to GAC over the last 10 years^4^, reflecting an emerging acceptance and need for GAC.^4^ There is a need for a systematic, evidence-based approach to health outcome measurement for analysis of treatment and cost-effectiveness, helping guide healthcare practices and health systems policies.

Health outcome measurement strategies include performance indicators, physiological parameters, provider-reported outcomes and patient-reported outcomes.^5^ There has been a historical overemphasis on performance indicators, physiological parameters and provider-reported outcomes and underemphasis on patient-reported outcomes.^6^ Patients’ perception of outcomes often differs from providers’ perception of the same outcome, calling into question whether current care is patient-centred. This is particularly relevant to GAC, where guidelines suggest that care should be patient-centred with care being provided to achieve patients’ rather than providers’ goals.^7 8^ GAC guidelines state that patient-reported outcomes must be used to understand patients’ goals and determine whether care provided is effectively meeting those goals.^9 10^ Patient-reported outcomes can be captured in a standardised and scalable way with patient-reported outcome measures (PROMs), which collect health information directly from patients.^11^

This systematic review aims to analyse health outcome measurement for GAC in Canada, identifying what is being measured, how it is being measured and barriers and enablers to both health outcome measurement and access to GAC. The rationale for focusing this review on Canada is that there is a paucity of published literature comprehensively assessing health outcome measurement for GAC in Canada. Further, Canada’s publicly funded healthcare system, characterised by provincial variability in access to gender-affirming interventions, presents a unique context for evaluating outcome measurement in GAC.^4^ Despite formalised policies supporting GAC, inconsistencies in outcome assessment persist across jurisdictions.^3 4^ Given the increasing emphasis on patient-reported outcomes in healthcare quality assessment,^11^ a focused review of the Canadian landscape allows for a comprehensive analysis of national trends, gaps and opportunities for standardisation. By restricting the scope to Canada, this review ensures the findings are directly applicable to Canadian healthcare policy and clinical practice, facilitating the development of a cohesive, evidence-based outcome measurement framework. The findings from this review offer a foundation for improving Canadian GAC practices.

## Methods

### Ethics

This is a systematic review and there is no primary data collection from human subjects; therefore, it was deemed exempt from ethical review.

### Patient and public involvement

This study includes patient and public involvement (PPI). Seven members representing the transgender and gender diverse community, ranging from young adults to adults, were recruited through contacting local charities (ie, Gender Identity Research and Education Society), online community transgender support groups and recruitment of one individual from an existing patient group with a separate research team. PPI were involved in providing feedback on the research question (ie, supporting the importance of reviewing outcome measurement in Canadian GAC). PPI meetings were held online over Microsoft Teams, where a meeting agenda was circulated prior to online meetings. An average of two to three meetings were held per year with regular email communication in the interim. During meetings, disagreements between PPI were resolved through discussion until all participants agreed on steps forward. PPI aided in identifying search terms, providing feedback on the relevance and importance of study findings and providing perspective based on personal experiences.

### Reflexivity and positionality

This review was conducted by a multidisciplinary team with diverse expertise in research methodology, systematic reviews, GAC and statistical analysis. The senior author (MD, DPhil) has a background in systematic review methodology and completed a doctoral dissertation at the University of Oxford focused on outcome measurement in GAC. As a member of the LGBTQ+ (Lesbian, Gay, Bisexual, Transgender, and Queer/Questioning) community, they bring both lived experience and academic expertise to this work. Another team member (MD candidate, PharmD, MSc) is also a member of the LGBTQ+community and has extensive experience conducting systematic reviews and research on GAC. A third team member (MD) is a clinician researcher who provides GAC in a clinical setting and has a research background in this area, contributing valuable practical insights. The final author (DPhil candidate) has expertise in research methodology, statistics and review articles, ensuring a rigorous analytical approach. We acknowledge that our collective identities, professional experiences and advocacy roles may shape our perspectives on GAC and outcome measurement. While this expertise strengthens our ability to critically evaluate the literature, it also introduces potential biases, such as a heightened awareness of structural barriers in healthcare and a commitment to advancing equitable and inclusive research. To mitigate these biases, we adhered to established systematic review methodologies, including predefined inclusion criteria, transparent data extraction and critical appraisal of study quality. We also engaged in ongoing reflexive discussions throughout the review process to ensure that interpretations were grounded in the data rather than personal assumptions. Furthermore, we recognise the limitations of existing research in this field, particularly the lack of racial and ethnic diversity in study samples. While this issue is highlighted in our discussion, we acknowledge that our own positionality as researchers with access to academic and medical institutions may influence how we perceive and frame these disparities.

### Study reporting

This systematic review follows Preferred Reporting Items for Systematic Reviews and Meta-Analyses (PRISMA) guidelines and was prospectively registered on PROSPERO (CRD42023462839).^12^

### Search strategy and selection process

The search strategy was developed by the research team and reviewed by a health sciences librarian. The search was conducted in five databases: MEDLINE, Embase, PsycINFO, Scopus and CINAHL to ensure comprehensive coverage of both peer-reviewed and grey literature. The searches covered literature from the inception of each database to the search date, 26 December 2023 with no language restrictions.

The subject headings and text words were chosen considering terms previously identified in published literature and terms in a published population search filter.^13^

The search strategy is available in online supplemental file 1.

### Eligibility criteria

The articles were included if they fulfilled the following inclusion criteria: (1) original article, (2) patients identifying as TGD and (3) patients accessing/receiving GAC in Canada. The articles were excluded if they did not fulfil the criteria.

### Selection and data collection process

The articles were exported to EndNote and then to Covidence for deduplication and screening. The title-abstract, full-text screening and data extraction occurred independently and in duplicate (LJ, RK, CC, MG) with conflicts resolved by a third reviewer (LJ).

### Data items

The team extracted detailed information from each article (see online supplemental appendix 1).

### Study risk of bias assessment

To assess the methodological quality of included studies, we used the Critical Appraisal Skills Programme (CASP) tool and the Joanna Briggs Institute (JBI) critical appraisal checklists.^14 15^ Two independent reviewers conducted the quality assessments, with discrepancies resolved through discussion or consultation with a third reviewer. Each study was evaluated using the tool most appropriate for its study design (eg, CASP for cohort studies research, JBI checklists for case reports). The scores were assigned based on predefined criteria, focusing on factors such as study design, risk of bias, sample representativeness and methodological rigour. All studies meeting the eligibility criteria were included in the review regardless of quality scores. While lower-quality studies were not excluded, their findings were interpreted with caution and their methodological limitations were explicitly considered in the synthesis. Studies with higher methodological rigour (eg, prospective designs, validated outcome measures and robust statistical analyses) were given greater interpretative weight in the discussion of results. Conversely, studies with high risk of bias or significant methodological concerns (eg, unclear inclusion criteria, small sample sizes or lack of validated outcome measures) were acknowledged as contributing to heterogeneity in findings.

### Synthesis methods

The Synthesis Without Meta-Analysis guideline was used to inform data synthesis reporting.^16^ The demographic and study information was analysed quantitatively through descriptive frequencies. Narrative synthesis was used to categorise barriers and enablers to outcome measurement.

## Results

### Study selection

**Figure 1:**
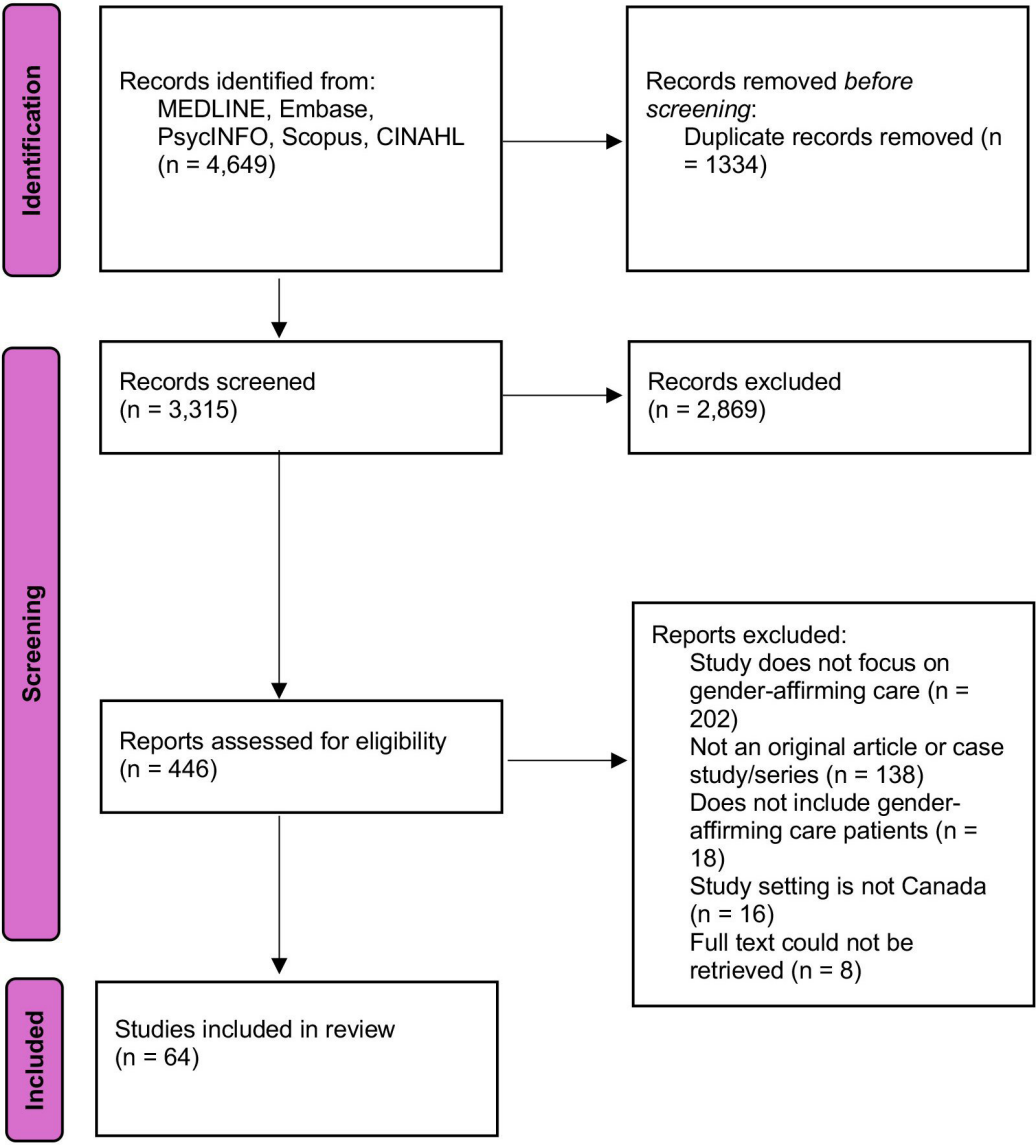
Preferred Reporting Items for Systematic Reviews and Meta-Analyses diagram of study selection.

The search resulted in 4649 articles, with 1334 duplicates removed, leaving 3315 articles. We excluded 2869 articles at the title and abstract screening stage and 382 articles at the full text screening stage, resulting in 64 included articles.^17^,^80^ This information is available in figure 1, the PRISMA diagram for this study and in online supplemental appendix 2.

### Patient characteristics

This systematic review represents a total of 6561 TGD patients. The mean age across articles ranged from 7.62 to 54.8 years with a total age range of 4.7–80 years. The race/ethnicity of TGD patients was reported in 22% (n=14) of studies, with most being white (38%, n=1039). The reporting of race and ethnicity was inconsistent across the 14 studies. Table 1 demonstrates data on race and ethnicity from these 14 studies.

**Table 1:** Race/ethnicity of participants represented (n=2754)

Race/ethnicity	Frequency, n (%)
Asian	79 (2.9)
Black	55 (2)
Black, African, Caribbean	4 (0.15)
Caribbean	1 (0.04)
Caucasian, Irish, Canadian, Quebecois	24 (0.87)
Chinese	1 (0.04)
East Asian	22 (0.8)
First Nation	46 (1.7)
Hispanic	3 (0.1)
Indigenous	142 (5.2)
Jewish	4 (0.15)
Latinx	42 (1.5)
Mixed	9 (0.3)
Non-First Nation	123 (4.5)
Non-Indigenous visible minority	20 (0.7)
Non-white or Indigenous	4 (0.15)
Other	30 (1.1)
Other or mixed	78 (2.8)
South Asian	41 (1.5)
Three or more	11 (0.4)
White	1039 (37.7)
NR	976 (35.4)

NRNot Reported

### Geographical locations represented

**Figure 2:**
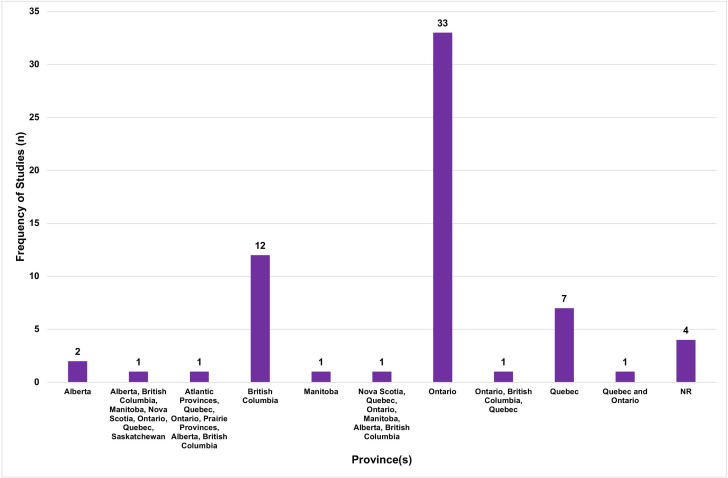
Province(s) represented among included articles. NR, not reported.

**Figure 3:**
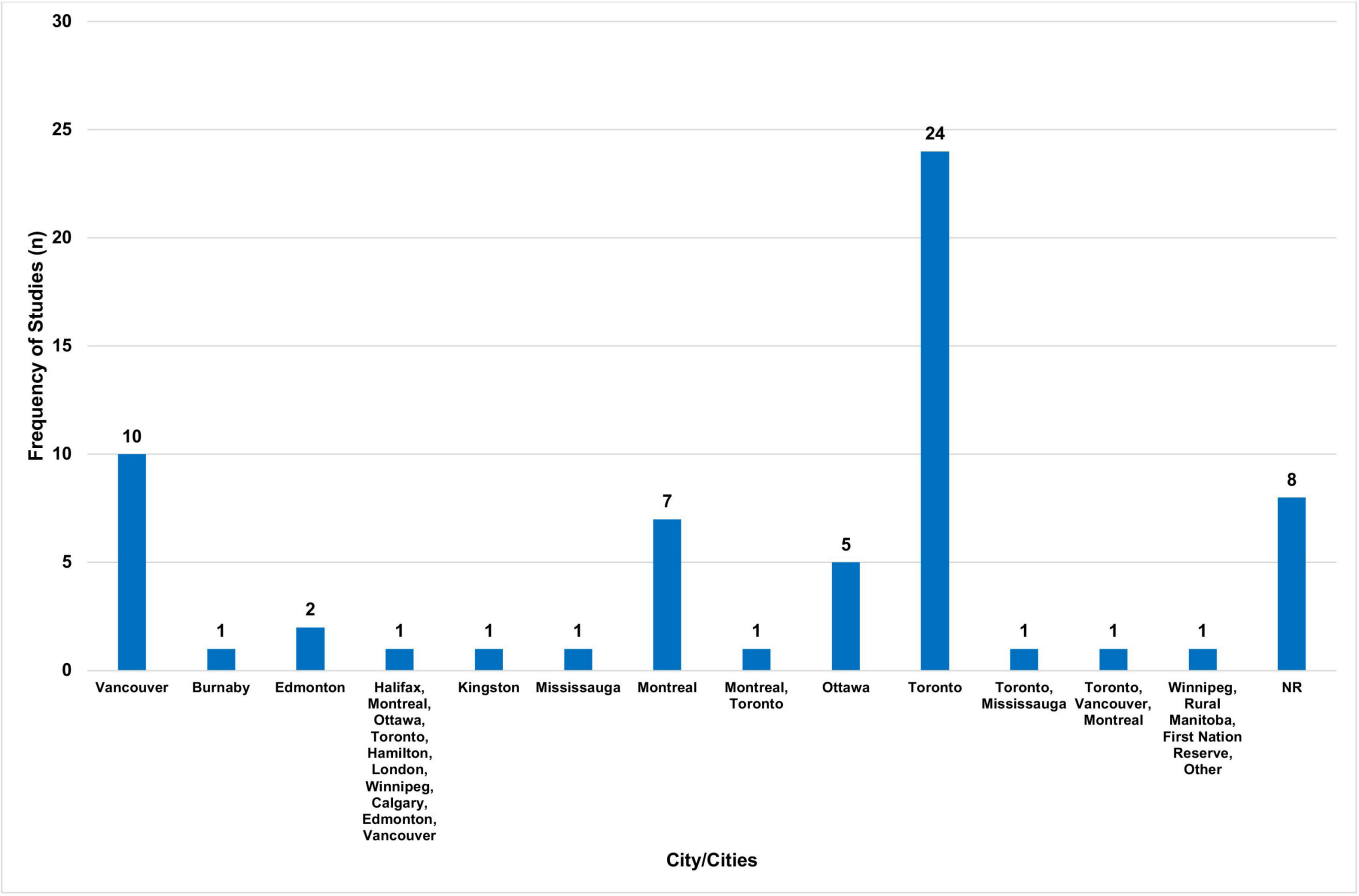
City/cities represented among included articles. NR, not reported.

Most studies were conducted in Ontario (n=33, 52%), British Columbia (n=12, 19%) and Quebec (n=7, 11%) (figure 2). The cities most represented were Toronto (n=24, 38%), Vancouver (n=10, 16%) and Montreal (n=7, 11%) (figure 3). The institutions most represented were the University of Toronto (n=27, 42%), University of British Columbia (n=13, 20%) and University of Ottawa (n=5, 8%). Online supplemental table 1 provides an overview of the locations represented in this study.

### Study information

The articles were published between 1979 and 2023. The publication rates have increased over time, peaking in 2023 (12 articles, 19%). Most were cohort studies (n=47, 73%). PPI was documented in two articles (3%). One (1.5%) sought input from community advisory councils and minority youth, while another (1.5%) included patient input for recruitment.^32 55^ Only one article (2%) provided a reflexivity statement.^68^ In total, 25 articles reported receiving funding. Table 2 provides an overview of study information.

**Table 2:** Study information for included articles

Year of publication	
Year	Frequency of papers, n (%)
1979	1 (1.6)
1985	2 (3.1)
1986	1 (1.6)
1987	1 (1.6)
1989	2 (3.1)
1995	1 (1.6)
2000	2 (3.1)
2007	1 (1.6)
2011	1 (1.6)
2014	2 (3.1)
2015	2 (3.1)
2016	4 (6.3)
2017	3 (4.7)
2018	8 (12.5)
2019	3 (4.7)
2020	4 (6.3)
2021	10 (15.6)
2022	4 (6.3)
2023	12 (18.8)
Study type	
Study type	Frequency
Case series/case report	12 (18.8)
Cohort study	47 (73.4)
Cross-sectional study	3 (4.7)
Mixed methods	1 (1.6)
Qualitative	1 (1.6)
Funding Information	
Funding	Frequency, n (%)
Yes	25 (39.1)
No	15 (23.4)
NR	24 (37.5)

NRNot Reported

### Gender-affirming care offered, and outcomes measured

**Figure 4:**
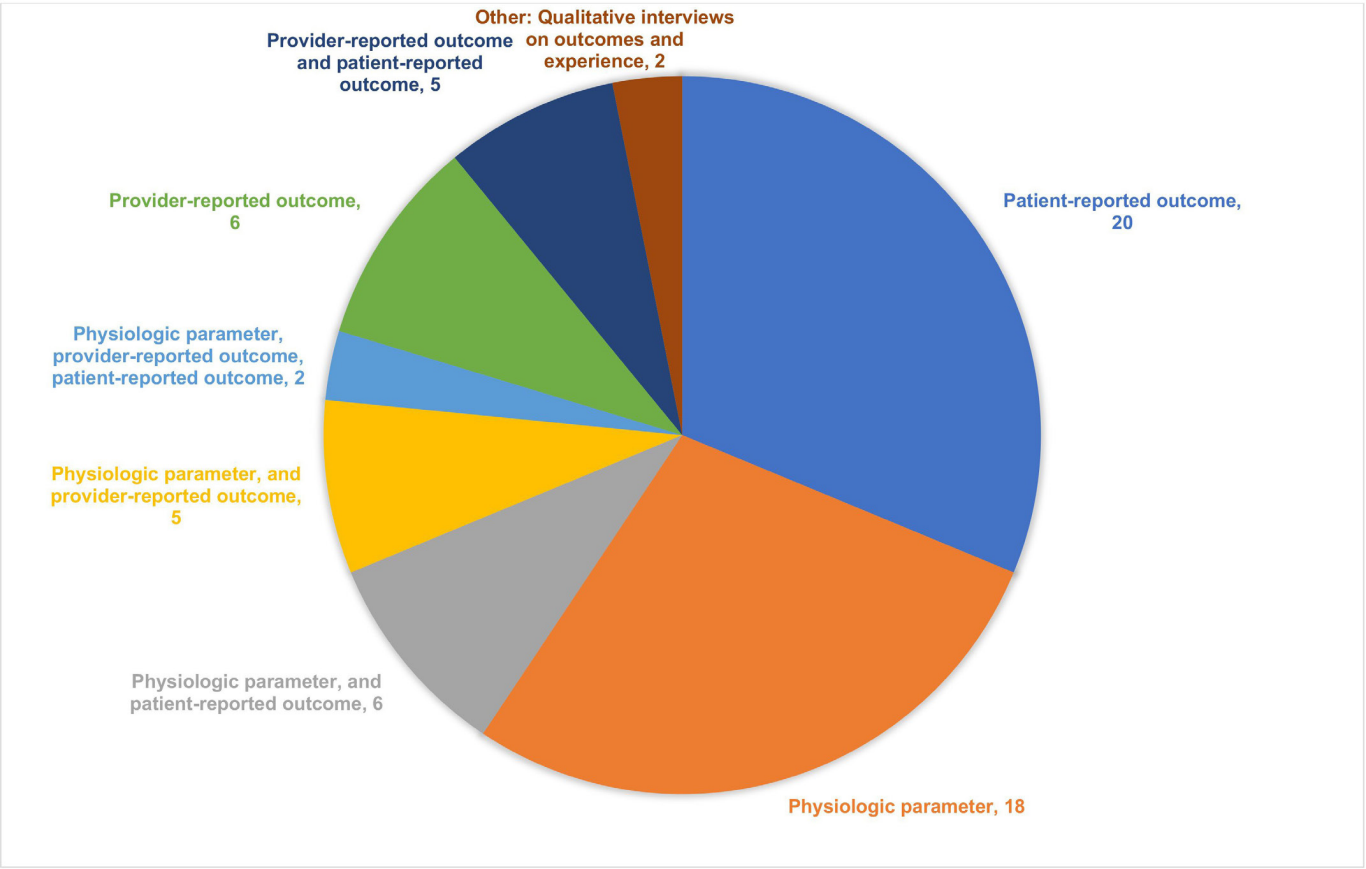
Outcome(s) measured among included articles.

The most common forms of GAC were hormonal (n=23, 36%), surgical (n=17, 27%) or both (n=8, 13%). The most common outcomes measured were patient-reported outcomes (n=20, 31%), physiological parameters (n=18, 28%), a combination of both (n=6, 9%) and provider-reported outcomes (n=6, 9%) (figure 4). Most studies did not administer a PROM (n=39, 61%). Physiological parameters were measured 85 times (some studies measured multiple physiological parameters) with the most common outcomes being serum testosterone (n=11, 13%), serum oestradiol (n=6, 7%), luteinising hormone (n=4, 5%) and follicle stimulating hormone (n=4, 5%). Provider-reported outcomes were measured 25 times, with the most common outcomes being postoperative complications (n=13, 52%), and provider assessment of photographs of the patient’s post-treatment (n=3, 12%). Table 3 provides an overview of GAC and outcome measures. Online supplemental tables 2 and 3 provide details on the physiologic and provider-reported outcomes measured.

**Table 3:** Overview of gender-affirming care provided and outcomes measured

Gender-affirming care	
Gender-affirming care provided	Frequency, n (%)
Hormonal (including puberty blockers)	23 (35.9)
Fertility preservation	3 (4.7)
Hormonal (including puberty blockers) and surgical	8 (12.5)
Hormonal (including puberty blockers) and voice therapy	2 (3.1)
Hormonal (including puberty blockers), psychological and surgery	3 (4.7)
Postoperative surgical care	1 (1.6)
Psychological	6 (9.4)
Surgical	17 (26.6)
Voice therapy	1 (1.6)
PROM administration	
Was a PROM administered?	Frequency
Yes	25 (39.1)
No	39 (60.9)*

* An ad hoc instrument was administered in 3three studies (4.7%).

PROMpatient-reported outcome measure

### Patient-reported outcome measures used for Canadian gender-affirming care

In total, 46 different PROMs were administered across the included articles.

24 articles provided information on the number of PROMs administered and frequency of PROMs administered: 10 articles administered only 1 PROM while 14 articles administered between 2 and 6 PROMs. Most articles administered a PROM only once (n=17, 71%). 23 articles provided information on the timing of PROM administration: pretreatment (n=6, 26%), post-treatment (n=8, 35%), pretreatment and post-treatment (n=7, 3%) or longitudinally (n=2, 9%). 21 articles provided information on the location of PROM administration, with most being in clinic (n=10, 48%). 11 articles provided information on the mode of PROM administration, most using electronic platforms (n=9, 82%). Only two articles discussed data security. Table 4 provides an overview of PROMs administered, data security considerations and frequency, timing, location and mode of administration.

**Table 4:** Overview of the number of PROMs administered, and frequency, timing, location and mode of administration

Number of PROMs administered (n=24 studies reporting this)	
Number of PROMs administered per study	Frequency, n (%)
1	10 (41.7)
2	1 (4.2)
3	5 (20.8)
4	3 (12.5)
5	3 (12.5)
6	2 (8.3)
Frequency of PROM administration (n=24 studies reporting this)	
Frequency of PROM administration	Frequency, n (%)
1	17 (70.8)
2	3 (12.5)
3	4 (16.7)
Timing of PROM administration (n=23 studies reporting this)	
When were PROMs administered?	Frequency, n (%)
Longitudinal	2 (8.7)
Post-treatment	8 (34.8)
Pretreatment	6 (26.1)
Pretreatment and post-treatment	7 (30.4)
Location of PROM completion (n=21 studies reporting this)	
Where were PROMs completed?	Frequency, n (%)
At home	8 (38.1)
In clinic	10 (47.6)
At home and in clinic	3 (14.3)
Mode of PROM administration (n=11 studies reporting this*)*	
Mode of PROM administration	Frequency, n (%)
Electronic, unspecified	5 (45.5)
Electronic on REDCap, or via paper booklets	1 (9.1)
Electronic on Qualtrics	3 (27.3)
iPad, pen and paper or email	1 (9.1)
Pen and paper	1 (9.1)
Data security considerations (n=2 studies reporting this)	
Data security	Frequency, n (%)
Secure, web-based server	1 (50)
Patients provided pseudonyms	1 (50)

PROMpatient-reported outcome measureREDCapResearch Electronic Data Capture

The mean number of items per PROM was 27 (SD=31), with a range of 1–126 items. PROMs measured a range of constructs, with psychosocial functioning being the most prominent (n=22, 48%). The most administered PROM was the Beck Depression Inventory. Online supplemental table 4 provides an overview of the PROMs administered, constructs measured, number of items per PROM and frequency of use.

### Barriers and enablers to gender-affirming care outcome measurement

Across all articles, none reported on enablers to outcome measurement in GAC. Among the articles that reported barriers (n=19, 30%), the most frequently discussed barriers pertained to PROMs, particularly the limited availability of validated PROMs capturing gender-related constructs.^49 59 70^ One article suggested that PROMs conceptualise gender in a binary manner, which may not be inclusive of non-binary patients.^30^ Some articles spoke to challenges with PROM completion, such as PROM completion by patients’ family rather than the patient themselves.^69^ Some articles spoke to limited PROM completion (ie, response rates) altogether.^49 50^ Some articles reported that outcomes were not measured in a standardised or systematic way, which may manifest in having limited baseline data for comparative purposes.^48 70^ These barriers were not discussed in-depth. There were reports of barriers pertaining to physiological parameters, with one article stating that serum hormone concentrations vary according to the timing of laboratory work relative to the timing of hormone administration, which is not standardised in practice.^19^ There were reports of barriers pertaining to provider-reported outcomes. One article discussed limitations of having providers assess outcomes through photographs, specifically the inability of a photograph to allow for assessment of skin quality.^51^

### Barriers and enablers to gender-affirming care access

Across all articles, none reported on enablers to GAC access. However, many reported on barriers (49, 77%). Some articles discussed stigma and discrimination, including TGD individuals experiencing negative encounters with healthcare professionals, particularly pronounced among patients facing multiple layers of stigma, such as HIV-related stigma.^50 55 75^ Some articles discussed lack of healthcare professional knowledge as a barrier resulting in a reluctance to provide or select treatments (particularly hormonal treatments).^36 49^ Some articles discussed geographical and socioeconomic barriers, including individuals travelling long distances to access GAC. Individuals incurred significant travel and time costs, leading to significant out-of-pocket expenses. Individuals also report that provincial health insurance covered some but not all medical services, contributing to out-of-pocket expenses.^21 51 69 70^ Some articles discussed psychosocial barriers, including instances where TGD individuals delayed or deferred care due to lack of societal acceptance and fear of coming out, which was particularly pronounced in racial and ethnic minorities, who were under-represented in GAC care clinics.^28 44 50 55^ Some articles discussed institutional barriers, including the use of cisnormative language in medical documentation and administration processes, which contributed to difficulty in accessing care.^55^ Some articles discussed protocol restrictions as a barrier, such as a need for parental consent.^68^

### Quality assessment and Oxford Level of Evidence

The CASP checklist was applied to 52 studies. All studies (n=52, 100%) addressed a clearly focused issue and recruited their cohorts in an acceptable way. Most studies (n=50, 96%) accurately measured both the exposure and outcome to minimise bias. Only 38% of studies identified all important confounding factors (n=20, 38%) and only 40% of studies accounted for confounding in design and/or analysis (n=21, 40%). Follow-up was complete and considered long enough in 77% of studies (n=40, 77%). See online supplemental table 5.

The JBI checklist was applied to 12 case reports/series. All reports (n=12, 100%) described patient demographics, history, current clinical condition, diagnostic tests, treatment procedures and post-intervention conditions. Most studies (n=9, 75%) described adverse events. See online supplemental table 6.

The Oxford Levels of Evidence were 2b for most (46, 72%), followed by 5 (12, 19%) and 2c (6, 9%).

## Discussion

This systematic review provides a comprehensive overview of health outcome measurement for GAC in Canada, highlighting what is being measured, how it is being measured and identifying barriers and enablers to both health outcome measurement and access to GAC. This information provides insights to clinicians, researchers, policymakers and commissioners aiming to improve outcome measurement in GAC.

The implementation of effective outcome measurement is essential to ensuring that care is responsive to patients’ needs. This systematic review revealed that while patient-reported outcomes were considered in~50% of studies, this is insufficient given the importance of capturing patient perspectives in GAC. When patient-reported outcomes were considered, PROMs were not always used, limiting the ability to compare data effectively. When PROMs were used, those most used did not capture gender-related concepts. Online supplemental table 4 provides a list of the PROMs used for GAC in Canada, organised by concepts measured. Inconsistencies in PROM selection, frequency, timing and location of administration also complicate this landscape, suggesting a need to standardise outcome measurement.

This systematic review identified several barriers to effective health outcome measurement in GAC. There is a limited availability of validated PROMs capturing gender-related constructs.^49 59 70^ The existing PROMs conceptualise gender in binary terms, excluding non-binary individuals, leading to incomplete or inaccurate assessments of patient outcomes.^30^ There are challenges with PROM completion, such as PROMs being completed by family members rather than patients.^69^

This systematic review identified several barriers to accessing GAC. Stigma and discrimination were major barriers, particularly pronounced in individuals facing multiple layers of stigma, such as those living with HIV.^50 55 75^ The impact of this stigma and discrimination was profound, resulting in a reluctance to access GAC. These findings are consistent with other studies that shed light on the detrimental effects of stigma on healthcare access for marginalised populations. Healthcare provider knowledge was another barrier, with many TGD individuals feeling that their healthcare providers did not possess the knowledge or skills necessary to address their needs.^36 49^ This resulted in a reluctance among healthcare providers to offer treatments. This finding underscores the need for comprehensive education for healthcare providers, ensuring that they are equipped to provide competent and compassionate care.

Geography and socioeconomic status were also barriers, with many TGD individuals travelling long distances to access specialised centres, incurring significant travel and time costs.^21 51 69 70^ This scarcity of such centres underscores the need for systems-level changes, such as increasing the number of specialised centres or enhancing access (eg, through virtual care). Psychosocial barriers, such as fear of societal non-acceptance, contributed to delays or deferrals of care.^28 44 50 55^ Institutional barriers, including the use of cisnormative language in medical documentation/administrative processes and protocol restrictions, such as the need for parental consent, also complicate access.^68^ These findings underscore the need to create more inclusive healthcare settings and systems, through practice and policy interventions that affirm identities of individuals.

This systematic review also identified a near absence of reflexivity and positionality statements. This is important, particularly in GAC, where bias and discrimination against TGD patients are substantial barriers to care. The inclusion of reflexivity and positionality statements aids in addressing these biases. This systematic review identified a near absence of PPI, which is contradictory to current best practices and may reduce the relevance of research findings. The inclusion of PPI is essential to ensure the relevance of research while enhancing trust with TGD individuals.

This systematic review also identified inconsistent reporting of race/ethnicity and gender across articles. The consistent reporting of these demographics is important for the identification of disparities in GAC. Gender should ideally be reported using the gold-standard two-step method, where patients are first asked their current gender identity and then asked their sex assigned at birth.^81^ The inconsistent reporting of race/ethnicity and gender results in a reduced ability to identify and address disparities, potentially perpetuating inequalities in care. Therefore, consistent demographic reporting is crucial to advancing equitable GAC. The studies included in this systematic review also predominantly focused on participants identified as white, highlighting a limitation in the current research landscape regarding GAC in Canada. This demographic homogeneity raises concerns about the representativeness of study populations and points to potential issues in data collection practices, where demographic variables such as race and ethnicity may not have been adequately captured. Alternatively, this skewed representation may reflect broader disparities in access to GAC, suggesting that black and brown patients face significant barriers to receiving care or participating in research studies. The lack of racial diversity in the study may mask critical differences in outcomes and experiences across diverse populations. Black and brown transgender and gender-diverse individuals often encounter unique challenges, including systemic discrimination, socioeconomic inequities and reduced access to healthcare services, all of which can influence both their healthcare experiences and outcomes. Therefore, the over-representation of white participants in the reviewed studies limits the generalisability of the findings and underscores the urgent need for more inclusive research practices. Addressing these disparities requires targeted efforts to improve demographic data collection, ensure equity in research participation and enhance access to GAC for all patients, irrespective of racial or ethnic background.

The quality assessment revealed considerable variability in study design, methodology and reporting. While some studies demonstrated strong methodological rigour, a significant proportion had notable limitations, including small sample sizes, retrospective designs and inconsistent use of validated PROMs. These findings highlight the need for more standardised and high-quality research in GAC, particularly regarding outcome measurement. The heterogeneity in study quality underscores challenges in synthesising evidence and reinforces the necessity for future research to adhere to rigorous methodological standards to improve the reliability and generalisability of findings. Despite these limitations, the inclusion of all studies, along with transparent quality assessment, provides a comprehensive overview of the existing literature and identifies critical gaps for future research.

Recommendations from this review include implementing PROMs, ideally ones capturing gender-related constructs, such as the Gender Congruence and Life Satisfaction Scale and the iTransQoL and validating them within the Canadian context.^82 83^ Standardising protocols for health outcome measurement will ensure that data collection is consistent and comparable across GAC settings. The Practical Guide to Implementing PROMs in GAC was developed in the UK and may find relevance in Canada, outlining evidence-based strategies to improve uptake of PROMs.^84^,^87^ There is a need to provide comprehensive education to healthcare providers on transgender health so that they are aware of best practice in GAC. There is a need to address geographical and socioeconomic barriers by increasing the number of specialised centres or increasing access to them; virtual care being one option. There is a need to encourage researchers to include reflexivity and positionality statements and collaborate with PPI.

While policies increasingly recognise the importance of GAC,^3 4 9 10^ there remains a gap in how outcomes are assessed and integrated into healthcare decision-making. Implementing a national strategy requires systemic, financial and institutional changes to ensure consistent and meaningful outcome measurement. One key step is the standardisation of PROMs in clinical practice. National regulatory bodies, such as the Canadian Medical Association and provincial health authorities, should establish guidelines mandating the use of validated PROMs in GAC. This would ensure consistency across provinces and facilitate comparisons between different healthcare settings, including in key settings such as paediatrics, medical imaging, pharmacy and endocrinology.^88^,^91^ Additionally, integrating PROMs into electronic health records would enable routine data collection, supporting both clinical decision-making and long-term outcome tracking. Investment in digital infrastructure and interoperability across provincial health systems is essential to facilitate this process. Financial support is crucial for implementation. The Canadian Institutes of Health Research and provincial health ministries should allocate dedicated funding for research and pilot programmes focused on PROM integration. Financial incentives for clinics and hospitals adopting standardised outcome measures could further encourage widespread implementation, along with transformational healthcare leadership.^92 93^ Additionally, insurance providers should explore reimbursement models linked to standardised outcome reporting, ensuring that PROMs play a role in funding decisions for gender-affirming interventions. Stakeholder engagement is essential to ensure that PROMs reflect the diverse needs of transgender and gender-diverse populations. Healthcare providers require training to administer and interpret PROMs effectively, with organisations such as the Canadian Professional Association for Transgender Health and the College of Family Physicians of Canada playing a central role in provider education. Equally important is the involvement of TGD individuals and advocacy groups in co-designing outcome measures to ensure relevance and inclusivity. Establishing a national research consortium on GAC outcomes, bringing together universities, policymakers and community organisations, would further support evidence-based policymaking.^94^

Canada can also learn from the UK’s national PROM strategy for GAC, which integrates standardised outcome measures into the National Health Service and tracks long-term patient-reported outcomes.^95^ A similar federated data-sharing system in Canada could enable outcome comparisons across provinces while protecting patient privacy. The UK model also emphasises longitudinal data collection, ensuring that patient experiences are captured beyond immediate post-treatment outcomes.^95^ Implementing a similar structure in Canada would require coordination between Health Canada, provincial ministries and research institutions to develop a unified framework. Standardised outcome measurement in GAC is critical for ensuring equitable, high-quality healthcare. Implementing a national strategy will require systemic reforms, financial investment and collaboration between healthcare providers, researchers and policymakers. By building on existing models, particularly the UK’s national approach, Canada has an opportunity to lead in evidence-based, patient-centred GAC. Future research should prioritise pilot studies to assess the feasibility of PROM integration in clinical settings, ensuring that outcome measurement informs both policy and patient care in a meaningful way.

This review underscores the critical need for standardised PROMs in GAC. In GAC, where the goal is not just procedural success but improvements in quality of life, social functioning and mental health, PROMs play an essential role in ensuring that care aligns with patient needs.^8 10^ Without standardised measures, it is difficult to assess long-term outcomes, track disparities or evaluate the effectiveness of interventions, limiting the ability to refine and improve care. For clinicians, integrating PROMs into practice ensures that patient perspectives are systematically incorporated into decision-making, allowing for more individualised and responsive care. Clinicians would benefit from training on how to administer and interpret PROMs effectively, ensuring they are used to guide discussions and treatment planning rather than simply collected as data points.^96^ PROMs also hold implications for healthcare systems, as standardised outcome measurement can inform funding decisions, service delivery and policy development.^95^ Clinics and hospitals adopting validated PROMs could be incentivised through funding models that prioritise outcome-based care, ensuring that gender-affirming services are supported at a structural level.

For patients, the implementation of PROMs has the potential to make care more patient-centred and transparent.^96^ When used effectively, PROMs can validate patient experiences and highlight areas where care needs improvement, ensuring that healthcare providers are accountable to the communities they serve.^96^ However, this requires careful attention to how PROMs are developed, ensuring that they are inclusive, reflective of diverse lived experiences and accessible across different populations. Beyond clinical applications, PROMs also have broader implications for policy and activism. Standardised outcome measurement can provide the data needed to advocate for improved access to GAC, demonstrating its impact on mental health and overall well-being. Policymakers can use PROM-driven evidence to justify expanded coverage, reduce wait times and allocate resources more effectively. Additionally, advocacy groups can leverage PROM data to push for healthcare reforms, ensuring that policies are informed by patient-reported experiences rather than assumptions or incomplete clinical metrics.^94^ The UK’s national PROM strategy for GAC offers a useful model, illustrating how systematic outcome measurement can drive evidence-based policy and care improvements.^95^

Standardising PROMs in GAC is not just a methodological concern but a necessary step toward ensuring that care is truly patient-centred, equitable and evidence-based. Implementation requires collaboration between healthcare providers, researchers, policymakers and patient communities to ensure that outcome measurement serves its intended purpose: improving care, reducing disparities and reinforcing the legitimacy of gender-affirming healthcare within broader medical and policy frameworks. Future research should focus on piloting PROMs in clinical settings, addressing barriers to their use and ensuring that they remain responsive to the evolving needs of the transgender and gender-diverse community.

It is important in PROM implementation to consider how licensing agreements can create barriers to their uptake.^97^ Proprietary PROMs often require institutions to enter licensing agreements that dictate how the tool can be used, distributed, and incorporated into clinical workflows. Even when proprietary PROMs are made available for non-profit use at no cost, these agreements can introduce administrative burdens, restrict integration and broader dissmination, creating barriers to implementation, especially in resource-limited settings. Beyond these logistical challenges, many proprietary PROMs require licensing fees for for-profit use, such as in industry sponsored trials. When PROM developers stand to collect licensing fees, there is an inherent conflict of interest where financial incentives may drive the development of tools that are comercially profitable rather than ones that best meet the needs of the patient population.^98^ As a result, outcome measurement can become shaped by industry-driven priorities as opposed to the broader needs of the healthcare systems and/or patients that interact with it. PROMs created using an equity-driven approach that are ready to implement include the Gender Congruence and Life Satisfaction Scale,^82^ and iTransQoL.^83^ These tools are already validated, widely accessible and capable of capturing key outcomes relevant to TGD populations. These tools are also concise in nature in regard to the number of items, which is a key consideration in PROM implementation to implement a PROM which is not burdensome for patients to complete.^96^ Using and/or optimising these tools for TGD-specific contexts represents a resource-conscious and actionable path forward to urgently improve outcome measurement in this area.

There are strengths and limitations of this study to consider. Strengths include collaboration with PPI, who confirmed the relevance of the research. Second, a comprehensive literature search was conducted without any restrictions on date or language, ensuring an inclusive review. Third, the study looked at all outcome measures used for GAC, providing a thorough understanding of the state of outcome measurements. Limitations include the fact that despite the comprehensive literature search, some articles might have been missed. In addition, this is a systematic review of published research, so some outcome measurement initiatives may not be represented. Further, due to the heterogeneity of included articles, a meta-analysis was unable to be performed and a narrative synthesis was conducted.

## Conclusion

This systematic review identifies health outcome measurement for GAC in Canada, including barriers to both GAC outcome measurement and GAC access. The underutilisation of PROMs is a gap in current practice, which can be addressed through the implementation of PROMs and ideally, ones that measure gender-related constructs. The development of standardised protocols for PROM implementation is also important. The implementation of these evidence-based practices will enhance the quality of care, ensure patient-centred outcomes and foster inclusive and supportive healthcare environments for TGD individuals in Canada.

## supplementary material

10.1136/bmjopen-2024-091135 online supplemental file 1

10.1136/bmjopen-2024-091135 online supplemental file 2

## Data Availability

All data relevant to the study are included in the article or uploaded as supplementary information.
