# Supplementary material for: Outcome measurement for gender-affirming care in Canada: a systematic review
Source: BMJ Open. 2025 Mar 12;15(3):e091135. doi: 10.1136/bmjopen-2024-091135 (PMC11904330; doi:10.1136/bmjopen-2024-091135)
Supplement: online supplemental file 1 [file bmjopen-15-3-s001.docx]

# Supplementary File 1: Search Strategies

## Medline Search Strategy

1. exp Gender Identity/
2. Gender ident*.mp.
3. exp Transgender Persons/
4. transgender*.mp.
5. transperson*.mp.
6. transpeople*.mp.
7. Trans gender*.mp.
8. exp Transsexualism/
9. Transexual*.mp.
10. Trans sexual*.mp.
11. Transman.mp.
12. Transmen.mp.
13. Trans man.mp.
14. Trans men.mp.
15. Transwoman.mp.
16. Transwomen.mp.
17. Trans woman.mp.
18. Trans women.mp.
19. Trans masc*.mp.
20. Transmasc*.mp.
21. Trans fem*.mp.
22. Transfem*.mp.
23. Gender quest*.mp.
24. Gender fluid*.mp.
25. Genderfluid*.mp.
26. Gender non-conforming.mp.
27. Gender nonconforming.mp.
28. Nonbinary.mp.
29. Agender*.mp.
30. Bigender*.mp.
31. Intergender*.mp.
32. Inter-gender*.mp.
33. Multigender.mp.
34. Multi-gender*.mp.
35. Genderqueer.mp.
36. Gender queer.mp.
37. Gender minority.mp.
38. Gender minorities.mp.
39. Gender divers*.mp.
40. Gender incongruent.mp.
41. Gender change.mp.
42. Trans adult*.mp.
43. Trans person*.mp.
44. Trans male*.mp.
45. Trans female*.mp.
46. Cross gender.mp.
47. Crossgender.mp.
48. exp Transvestism/
49. Transvest*.mp.
50. Cross dress*.mp.
51. two-spirit*.mp.
52. Gender affirm*.mp.
53. exp Gender Dysphoria/
54. gender dysphor*.mp.
55. Gender euphoria.mp.
56. Gender identity disorder*.mp.
57. exp Health Services for Transgender Persons/
58. exp Sex Reassignment Procedures/
59. Sex* reassignment*.mp.
60. Gender reassign*.mp.
61. Gender confirm*.mp.
62. Sex* reversal*.mp.
63. Sex* transition*.mp.
64. Sex* change*.mp.
65. Sex* conform*.mp.
66. Sex* affirm*.mp.
67. Genital reconstruct*.mp.
68. Recon* surg*.mp.
69. Cross-sex hormone*.mp.
70. Cross-gender hormone*.mp.
71. Gonadotropin-Releasing Hormone/
72. Gonadotropin releasing hormone agonist*.mp.
73. GnRH agonist*.mp.
74. exp Gonadal Steroid Hormones/
75. Androgen agonist*.mp.
76. Hormon* therap*.mp.
77. exp Estrogen Replacement Therapy/
78. Estrogen therap*.mp.
79. Estrogen replace*.mp.
80. Anti-estrogen*.mp.
81. Estrogen antagonist*.mp.
82. exp Estrogen Antagonists/
83. Estrogen blocker*.mp.
84. exp Testosterone/
85. testosterone therap*.mp.
86. testosterone replace*.mp.
87. exp Androgen Antagonists/
88. Androgen antagonist*.mp.
89. Anti-androgen*.mp.
90. Androgen blocker*.mp.
91. exp Mastectomy/
92. Mastectom*.mp.
93. Breast reconstruct*.mp.
94. exp Mammaplasty/
95. Mammaplast*.mp.
96. Breast endoprosthesis.mp.
97. Breast implant*.mp.
98. Tissue expan*.mp.
99. Thorax surger*.mp.
100. Chest contour*.mp.
101. Chest-wall contour*.mp.
102. Chest reconstruct*.mp.
103. Bottom surg*.mp.
104. Penile reconstruct*.mp.
105. Penile construct*.mp.
106. Testic* construct*.mp.
107. Testicular implant*.mp.
108. exp Castration/
109. Orchiectom*.mp.
110. Vaginoplasty.mp.
111. Vagina* reconstruct*.mp.
112. Vagina* construct*.mp.
113. Penectomy.mp.
114. Metoidioplasty.mp.
115. Phalloplasty.mp.
116. penile implant*.mp.
117. Genitoplasty.mp.
118. Psych* support*.mp.
119. Psych* care.mp.
120. exp Physical Therapy Modalities/
121. Physical therap*.mp.
122. Physiotherap*.mp.
123. exp Occupational Therapy/
124. Occupational therap*.mp.
125. exp Voice Training/
126. Voice training.mp.
127. exp Psychotherapy/
128. Psychotherap*.mp.
129. Counselling.mp.
130. exp Mental Health Services/
131. exp Community Health Services/
132. exp Psychosocial Intervention/
133. Mental health service*.mp.
134. Community health service*.mp.
135. Psychosocial intervention*.mp.
136. exp Speech Therapy/
137. Speech therap*.mp.
138. Voice therap*.mp.
139. Voice surg*.mp.
140. tracheal cartilage shave.mp.
141. otorhinolaryngolog* surg*.mp.
142. Gonadectomy.mp.
143. Voice mod*.mp.
144. exp Feminization/
145. Feminiz*.mp.
146. Masculinization.mp.
147. exp Virilism/
148. Androgenization.mp.
149. Penile implantation*.mp.
150. Top surgery.mp.
151. exp Orchiectomy/
152. exp Penile Implantation/
153. exp Hysterectomy/
154. exp Hormone Replacement Therapy/
155. FTM Patients.mp.
156. MTF Patients.mp.
157. Male to female transgender.mp.
158. Female to male transgender.mp.
159. MTF.mp.
160. FTM.mp.
161. 1 or 2 or 3 or 4 or 5 or 6 or 7 or 8 or 9 or 10 or 11 or 12 or 13 or 14 or 15 or 16 or 17 or 18 or 19 or 20 or 21 or 22 or 23 or 24 or 25 or 26 or 27 or 28 or 29 or 30 or 31 or 32 or 33 or 34 or 35 or 36 or 37 or 38 or 39 or 40 or 41 or 42 or 43 or 44 or 45 or 46 or 47 or 48 or 49 or 50 or 51 or 52 or 53 or 54 or 55 or 56 or 155 or 156 or 157 or 158 or 159 or 160
162. 57 or 58 or 59 or 60 or 61 or 62 or 63 or 64 or 65 or 66 or 67 or 68 or 69 or 70 or 71 or 72 or 73 or 74 or 75 or 76 or 77 or 78 or 79 or 80 or 81 or 82 or 83 or 84 or 85 or 86 or 87 or 88 or 89 or 90 or 91 or 92 or 93 or 94 or 95 or 96 or 97 or 98 or 99 or 100 or 101 or 102 or 103 or 104 or 105 or 106 or 107 or 108 or 109 or 110 or 111 or 112 or 113 or 114 or 115 or 116 or 117 or 118 or 119 or 120 or 121 or 122 or 123 or 124 or 125 or 126 or 127 or 128 or 129 or 130 or 131 or 132 or 133 or 134 or 135 or 136 or 137 or 138 or 139 or 140 or 141 or 142 or 143 or 144 or 145 or 146 or 147 or 148 or 149 or 150 or 151 or 152 or 153 or 154
163. (Canad* or British Columbia or Colombie Britannique or Alberta* or Saskatchewan or Manitoba* or Ontario or Quebec or (New Brunswick not New Jersey) or Nouveau Brunswick or Nova Scotia or Nouvelle Ecosse or Prince Edward Island or Newfoundland or Labrador or Nunavut or NWT or Northwest Territories or Yukon or Nunavik or Inuvialuit).mp,jw,nw,in. or (Abbotsford or Airdrie or Ajax or Aurora or Barrie or Belleville or Blainville or Brampton or Brantford or Brossard or Burlington or Burnaby or Caledon or Calgary or Cape Breton or Chatham Kent or Chilliwack or Clarington or Coquitlam or Drummondville or Edmonton or Fredericton or Fort McMurray or Gatineau or Granby or Grande Prairie or Sudbury or Guelph or Halton Hills or Iqaluit or Inuvik or Kamloops or Kawartha Lakes or Kelowna or Kingston or Kitchener or Langley or Laval or Lethbridge or Levis or Longueuil or Maple Ridge or Markham or Medicine Hat or Milton or Mirabel or Mississauga or Moncton or Montreal or Nanaimo or New Westminster or Newmarket or Niagara Falls or Norfolk County or North Bay or North Vancouver or Oakville or Oshawa or Ottawa or Peterborough or Pickering or Port Coquitlam or Prince George or Quebec City or Red Deer or Regina or Repentigny or Richmond or Richmond Hill or Saanich or Saguenay or Saint John or Saint-Hyacinthe or Saint-Jean-sur-Richelieu or Saint-Jerome or Sarnia or Saskatoon or Sault Ste Marie or Sherbrooke or St Albert or St Catharines or St John's or Strathcona County or Surrey or Terrebonne or Thunder Bay or Toronto or Trois-Rivieres or Vancouver or Vaughan or ((Cambridge or (Halifax or Hamilton or London or Victoria or Waterloo or Welland or Whitby or Windsor)) not (UK or Britain or United Kingdom or England or Australia)) or Whitehorse or Winnipeg or Wood Buffalo or Yellowknife).ti,ab,kw,in.
164. ("Concordia University" or "Université du Québec à Chicoutimi" or "Université du Québec à Montréal" or "Université du Québec en Abitibi-Témiscamingue" or "Université du Québec à Trois-Rivières" or "Université du Québec en Outaouais" or "Université du Québec" or "Brock University" or "Concordia Lutheran Theological Seminary" or "Carleton University" or "Dominican University College" or "University of Guelph" or "Lakehead University" or "Laurentian University of Sudbury" or "McMaster University" or "Nipissing University" or "University of Ottawa" or "Saint-Paul University" or "Queen's University").in.
165. ("Memorial University of Newfoundland" or "University of Prince Edward Island" or "Acadia University" or "Acadia Divinity College" or "Atlantic School of Theology" or "Cape Breton University" or "Dalhousie University" or "University of King's College" or "Mount Saint Vincent University" or "Nova Scotia College of Art and Design University" or "Université Sainte-Anne" or "St. Francis Xavier University" or "Saint Mary's University" or "Mount Allison University" or "University of New Brunswick" or "Université de Moncton" or "St. Thomas University" or "Bishop's University" or "McGill University").in.
166. ("Ryerson University" or "University of Toronto" or "St. Augustine's Seminary" or "University of St. Michael's College" or "University of Trinity College" or "Victoria University" or "Knox College" or "Wycliffe College" or "Regis College" or "Trent University" or "University of Waterloo" or "St. Jerome's University" or "Renison University College" or "Conrad Grebel University College" or "University of Western Ontario" or "Brescia University College" or "Huron University College" or "King's College" or "Wilfred Laurier University").in.
167. ("University of Windsor" or "York University" or "Ontario College of Art" or "University of Ontario Institute of Technology" or "Algoma University College" or "University of Sudbury" or "Université de Hearst" or "Huntington University" or "Thorneloe University" or "Brandon University" or "Canadian Mennonite University" or "University of Manitoba" or "Université de Saint-Boniface" or "St. Andrew's College" or "University of Winnipeg" or "University of Regina" or "Campion College" or "Luther College" or "University of Saskatchewan").in.
168. ("College of Emmanuel and St. Chad" or "Lutheran Theological Seminary" or "St. Andrew's College" or "St. Thomas More College" or "Horizon College & Seminary" or "University of Alberta" or "Athabasca University" or "University of Calgary" or "Burman University" or "Concordia University of Edmonton" or "University of Lethbridge" or "The King's University College" or "Ambrose University" or "Grant MacEwan University" or "Mount Royal University" or "University of British Columbia" or "University of Northern British Columbia" or "Royal Roads University" or "Simon Fraser University").in.
169. ("University of Victoria" or "Thompson Rivers University" or "Capilano University" or "Vancouver Island University" or "Emily Carr University of Art and Design" or "Kwantlen Polytechnic University" or "University of the Fraser Valley" or "Yukon University").in.
170. 164 or 165 or 166 or 167 or 168 or 169
171. 163 or 170
172. 161 and 162 and 171

## PsychINFO Search Strategy

1. exp gender identity/
2. exp transgender/
3. exp transsexualism/
4. exp transvestism/
5. exp gender dysphoria/
6. exp gender reassignment/
7. exp gonadotropic hormones/
8. exp sex hormones/
9. exp hormone therapy/
10. exp Antiestrogens/
11. exp testosterone/
12. exp antiandrogens/
13. exp mastectomy/
14. exp castration/
15. exp physical therapy/
16. exp occupational therapy/
17. exp psychotherapy/
18. exp mental health services/
19. exp community mental health services/
20. exp psychosocial interventions/
21. exp speech therapy/
22. exp hysterectomy/
23. exp hormone therapy/
24. (Gender ident* or transgender* or transperson* or transpeople* or Trans gender* or Transexual* or Trans sexual* or Transman or Transmen or Trans man or Trans men or Transwoman or Transwomen or Trans woman or Trans women or Trans masc* or Transmasc* or Trans fem* or Transfem* or Gender quest* or Gender fluid* or Genderfluid* or Gender non-conforming or Gender nonconforming or Nonbinary or Agender* or Bigender* or Intergender* or Inter-gender* or Multigender or Multi-gender* or Genderqueer or Gender queer or Gender minority or Gender minorities or Gender divers* or Gender incongruent or Gender change or Trans adult* or Trans person* or Trans male* or Trans female* or Cross gender or Crossgender or Transvest* or Cross dress* or two-spirit* or Gender affirm* or gender dysphor* or Gender euphoria or Gender identity disorder* or MTF Patients or Male to female transgender or Female to male transgender or MTF or FTM).mp.
25. (Sex* reassignment* or Gender reassign* or Gender confirm* or Sex* reversal* or Sex* transition* or Sex* change* or Sex* conform* or Sex* affirm* or Genital reconstruct* or Recon* surg* or Cross-sex hormone* or Cross-gender hormone* or Gonadotropin releasing hormone agonist* or GnRH agonist* or Androgen agonist* or Hormon* therap* or Estrogen therap* or Estrogen replace* or Anti-estrogen* or Estrogen antagonist* or Estrogen blocker* or Testosterone therap* or Testosterone replace* or Androgen antagonist* or Anti-androgen* or Androgen blocker* or Mastectom* or Breast reconstruct* or Mammaplast* or Breast endoprosthesis or Breast implant* or Tissue expan* or Thorax surger* or Chest contour* or Chest-wall contour* or Chest reconstruct* or Bottom surg* or Penile reconstruct* or Penile construct* or Testic* construct* or Testicular implant* or Orchiectom* or Vaginoplasty or Vagina* reconstruct* or Vagina* construct* or Penectomy or Metoidioplasty or Phalloplasty or Penile implant* or Genitoplasty or Psych* support* or Psych* care or Physical therap* or Physiotherap* or Occupational therap* or Voice training or Psychotherap* or Counselling or Mental health service* or Community health service* or Psychosocial intervention* or Speech therap* or Voice therap* or Voice surg* or tracheal cartilage shave or otorhinolaryngolog* surg* or Gonadectomy or Voice mod* or Feminiz* or Masculinization or Androgenization or Penile implantation* or Top surgery).mp.
26. (Canad* or British Columbia or Colombie Britannique or Alberta* or Saskatchewan or Manitoba* or Ontario or Quebec or (New Brunswick not New Jersey) or Nouveau Brunswick or Nova Scotia or Nouvelle Ecosse or Prince Edward Island or Newfoundland or Labrador or Nunavut or NWT or Northwest Territories or Yukon or Nunavik or Inuvialuit).mp,jw,in. or (Abbotsford or Airdrie or Ajax or Aurora or Barrie or Belleville or Blainville or Brampton or Brantford or Brossard or Burlington or Burnaby or Caledon or Calgary or Cape Breton or Chatham Kent or Chilliwack or Clarington or Coquitlam or Drummondville or Edmonton or Fredericton or Fort McMurray or Gatineau or Granby or Grande Prairie or Sudbury or Guelph or Halton Hills or Iqaluit or Inuvik or Kamloops or Kawartha Lakes or Kelowna or Kingston or Kitchener or Langley or Laval or Lethbridge or Levis or Longueuil or Maple Ridge or Markham or Medicine Hat or Milton or Mirabel or Mississauga or Moncton or Montreal or Nanaimo or New Westminster or Newmarket or Niagara Falls or Norfolk County or North Bay or North Vancouver or Oakville or Oshawa or Ottawa or Peterborough or Pickering or Port Coquitlam or Prince George or Quebec City or Red Deer or Regina or Repentigny or Richmond or Richmond Hill or Saanich or Saguenay or Saint John or Saint-Hyacinthe or Saint-Jean-sur-Richelieu or Saint-Jerome or Sarnia or Saskatoon or Sault Ste Marie or Sherbrooke or St Albert or St Catharines or St John's or Strathcona County or Surrey or Terrebonne or Thunder Bay or Toronto or Trois-Rivieres or Vancouver or Vaughan or ((Cambridge or (Halifax or Hamilton or London or Victoria or Waterloo or Welland or Whitby or Windsor)) not (UK or Britain or United Kingdom or England or Australia)) or Whitehorse or Winnipeg or Wood Buffalo or Yellowknife).mp,in.
27. ("Concordia University" or "Université du Québec à Chicoutimi" or "Université du Québec à Montréal" or "Université du Québec en Abitibi-Témiscamingue" or "Université du Québec à Trois-Rivières" or "Université du Québec en Outaouais" or "Université du Québec" or "Brock University" or "Concordia Lutheran Theological Seminary" or "Carleton University" or "Dominican University College" or "University of Guelph" or "Lakehead University" or "Laurentian University of Sudbury" or "McMaster University" or "Nipissing University" or "University of Ottawa" or "Saint-Paul University" or "Queen's University").in.
28. ("Memorial University of Newfoundland" or "University of Prince Edward Island" or "Acadia University" or "Acadia Divinity College" or "Atlantic School of Theology" or "Cape Breton University" or "Dalhousie University" or "University of King's College" or "Mount Saint Vincent University" or "Nova Scotia College of Art and Design University" or "Université Sainte-Anne" or "St. Francis Xavier University" or "Saint Mary's University" or "Mount Allison University" or "University of New Brunswick" or "Université de Moncton" or "St. Thomas University" or "Bishop's University" or "McGill University").in.
29. ("Ryerson University" or "University of Toronto" or "St. Augustine's Seminary" or "University of St. Michael's College" or "University of Trinity College" or "Victoria University" or "Knox College" or "Wycliffe College" or "Regis College" or "Trent University" or "University of Waterloo" or "St. Jerome's University" or "Renison University College" or "Conrad Grebel University College" or "University of Western Ontario" or "Brescia University College" or "Huron University College" or "King's College" or "Wilfred Laurier University").in.
30. ("University of Windsor" or "York University" or "Ontario College of Art" or "University of Ontario Institute of Technology" or "Algoma University College" or "University of Sudbury" or "Université de Hearst" or "Huntington University" or "Thorneloe University" or "Brandon University" or "Canadian Mennonite University" or "University of Manitoba" or "Université de Saint-Boniface" or "St. Andrew's College" or "University of Winnipeg" or "University of Regina" or "Campion College" or "Luther College" or "University of Saskatchewan").in.
31. ("College of Emmanuel and St. Chad" or "Lutheran Theological Seminary" or "St. Andrew's College" or "St. Thomas More College" or "Horizon College & Seminary" or "University of Alberta" or "Athabasca University" or "University of Calgary" or "Burman University" or "Concordia University of Edmonton" or "University of Lethbridge" or "The King's University College" or "Ambrose University" or "Grant MacEwan University" or "Mount Royal University" or "University of British Columbia" or "University of Northern British Columbia" or "Royal Roads University" or "Simon Fraser University").in.
32. ("University of Victoria" or "Thompson Rivers University" or "Capilano University" or "Vancouver Island University" or "Emily Carr University of Art and Design" or "Kwantlen Polytechnic University" or "University of the Fraser Valley" or "Yukon University").in.
33. 1 or 2 or 3 or 4 or 5 or 24
34. 6 or 7 or 8 or 9 or 10 or 11 or 12 or 13 or 14 or 15 or 16 or 17 or 18 or 19 or 20 or 21 or 22 or 23 or 25
35. 26 or 27 or 28 or 29 or 30 or 31 or 32
36. 33 and 34 and 35

## SCOPUS Search Strategy

( ( ( ALL ( canad* OR "british columbia" OR "colombie britannique" OR alberta* OR saskatchewan OR manitoba* OR ontario OR quebec OR "new brunswick" OR "nouveau brunswick" OR "nova scotia" OR "nouvelle ecosse" OR "prince edward island" OR newfoundland OR labrador OR nunavut OR nwt OR "northwest territories" OR yukon OR nunavik OR inuvialuit OR abbotsford OR airdrie OR ajax OR aurora OR barrie OR belleville OR blainville OR brampton OR brantford OR brossard OR burlington OR burnaby OR caledon OR calgary OR cambridge OR "cape breton" OR chatham OR kent OR chilliwack OR clarington OR coquitlam OR drummondville OR edmonton OR "fort mcmurray" OR fredericton OR gatineau OR granby OR "grande prairie" OR sudbury OR guelph OR "halton hills" OR iqaluit OR inuvik OR kamloops OR "kawartha lakes" OR kelowna OR kingston OR kitchener OR langley OR laval OR lethbridge OR levis OR longueuil OR "maple ridge" OR markham OR "medicine hat" OR milton OR mirabel OR mississauga OR moncton OR montreal OR nanaimo OR "new westminster" OR newmarket OR "niagara falls" OR "norfolk county" OR "north bay" OR "north vancouver" OR oakville OR oshawa OR ottawa OR peterborough OR pickering OR "port coquitlam" OR "prince george" OR "quebec city" OR "red deer" OR regina OR repentigny OR richmond OR "richmond hill" OR saanich OR saguenay OR "saint john" OR "saint-hyacinthe" OR "saint-jean-sur-richelieu" OR "saint-jerome" OR sarnia OR saskatoon OR "sault ste marie" OR sherbrooke OR "st albert" OR "st catharines" OR "st john&apos;s" OR "strathcona county" OR surrey OR terrebonne OR "thunder bay" OR toronto OR "trois-rivieres" OR vancouver OR vaughan OR halifax OR hamilton OR london OR victoria OR waterloo OR welland OR whitby OR windsor OR whitehorse OR winnipeg OR "wood buffalo" OR yellowknife ) AND NOT ( ALL ( uk OR "united kingdom" OR britain OR england OR australia OR "united states" OR virginia OR "new jersey" ) ) ) ) OR ( AFFIL ( ( "concordia university" OR "universit&amp;eacute; du qu&amp;eacute;bec &amp;agrave; chicoutimi" OR "universit&amp;eacute; du qu&amp;eacute;bec &amp;agrave; montr&amp;eacute;al" OR "universit&amp;eacute; du qu&amp;eacute;bec en abitibi-t&amp;eacute;miscamingue" OR "universit&amp;eacute; du qu&amp;eacute;bec &amp;agrave; trois-rivi&amp;egrave;res" OR "universit&amp;eacute; du qu&amp;eacute;bec en outaouais" OR "universit&amp;eacute; du qu&amp;eacute;bec" OR "brock university" OR "concordia lutheran theological seminary" OR "carleton university" OR "dominican university college" OR "university of guelph" OR "lakehead university" OR "laurentian university of sudbury" OR "mcmaster university" OR "nipissing university" OR "university of ottawa" OR "saint-paul university" OR "queen&apos;s university" ) OR ( "memorial university of newfoundland" OR "university of prince edward island" OR "acadia university" OR "acadia divinity college" OR "atlantic school of theology" OR "cape breton university" OR "dalhousie university" OR "university of king&apos;s college" OR "mount saint vincent university" OR "nova scotia college of art and design university" OR "universit&amp;eacute; sainte-anne" OR "st. francis xavier university" OR "saint mary&apos;s university" OR "mount allison university" OR "university of new brunswick" OR "universit&amp;eacute; de moncton" OR "st. thomas university" OR "bishop&apos;s university" OR "mcgill university" ) OR ( "ryerson university" OR "university of toronto" OR "st. augustine&apos;s seminary" OR "university of st. michael&apos;s college" OR "university of trinity college" OR "victoria university" OR "knox college" OR "wycliffe college" OR "regis college" OR "trent university" OR "university of waterloo" OR "st. jerome&apos;s university" OR "renison university college" OR "conrad grebel university college" OR "university of western ontario" OR "brescia university college" OR "huron university college" OR "king&apos;s college" OR "wilfred laurier university" ) OR ( "university of windsor" OR "york university" OR "ontario college of art" OR "university of ontario institute of technology" OR "algoma university college" OR "university of sudbury" OR "universit&amp;eacute; de hearst" OR "huntington university" OR "thorneloe university" OR "brandon university" OR "canadian mennonite university" OR "university of manitoba" OR "universit&amp;eacute; de saint-boniface" OR "st. andrew&apos;s college" OR "university of winnipeg" OR "university of regina" OR "campion college" OR "luther college" OR "university of saskatchewan" ) OR ( "college of emmanuel and st. chad" OR "lutheran theological seminary" OR "st. andrew&apos;s college" OR "st. thomas more college" OR "horizon college &amp; seminary" OR "university of alberta" OR "athabasca university" OR "university of calgary" OR "burman university" OR "concordia university of edmonton" OR "university of lethbridge" OR "the king&apos;s university college" OR "ambrose university" OR "grant macewan university" OR "mount royal university" OR "university of british columbia" OR "university of northern british columbia" OR "royal roads university" OR "simon fraser university" ) OR ( "university of victoria" OR "thompson rivers university" OR "capilano university" OR "vancouver island university" OR "emily carr university of art and design" OR "kwantlen polytechnic university" OR "university of the fraser valley" OR "yukon university" ) . ) ) ) AND ( ( TITLE-ABS-KEY ( "gender ident*" OR "transgender*" OR "transperson*" OR "transpeople*" OR "trans gender*" OR "transexual*" OR "trans sexual*" OR "transman" OR "transmen" OR "trans man" OR "trans men" OR "transwoman" OR "transwomen" OR "trans woman" OR "trans women" OR "trans masc*" OR "transmasc*" OR "trans fem*" OR "transfem*" OR "gender quest*" OR "gender fluid*" OR "genderfluid*" OR "gender non-conforming" OR "gender nonconforming" OR "nonbinary" OR "agender*" OR "bigender*" OR "intergender*" OR "inter-gender*" OR "multigender" OR "multi-gender*" OR "genderqueer" OR "gender queer" OR "gender minority" OR "gender minorities" OR "gender divers*" OR "gender incongruent" OR "gender change" OR "trans adult*" OR "trans person*" OR "trans male*" OR "trans female*" OR "cross gender" OR "crossgender" OR "transvest*" OR "cross dress*" OR "two-spirit*" OR "gender affirm*" OR "gender dysphor*" OR "gender euphoria" OR "gender identity disorder*" OR "mtf patients" OR "male to female transgender" OR "female to male transgender" OR "mtf" OR "ftm" ) AND TITLE-ABS-KEY ( "sex* reassignment*" OR "gender reassign*" OR "gender confirm*" OR "sex* reversal*" OR "sex* transition*" OR "sex* change*" OR "sex* conform*" OR "sex* affirm*" OR "genital reconstruct*" OR "recon* surg*" OR "cross-sex hormone*" OR "cross-gender hormone*" OR "gonadotropin releasing hormone agonist*" OR "gnrh agonist*" OR "androgen agonist*" OR "hormon* therap*" OR "estrogen therap*" OR "estrogen replace*" OR "anti-estrogen*" OR "estrogen antagonist*" OR "estrogen blocker*" OR "testosterone therap*" OR "testosterone replace*" OR "androgen antagonist*" OR "anti-androgen*" OR "androgen blocker*" OR "mastectom*" OR "breast reconstruct*" OR "mammaplast*" OR "breast endoprosthesis" OR "breast implant*" OR "tissue expan*" OR "thorax surger*" OR "chest contour*" OR "chest-wall contour*" OR "chest reconstruct*" OR "bottom surg*" OR "penile reconstruct*" OR "penile construct*" OR "testic* construct*" OR "testicular implant*" OR "orchiectom*" OR "vaginoplasty" OR "vagina* reconstruct*" OR "vagina* construct*" OR "penectomy" OR "metoidioplasty" OR "phalloplasty" OR "penile implant*" OR "genitoplasty" OR "psych* support*" OR "psych* care" OR "physical therap*" OR "physiotherap*" OR "occupational therap*" OR "voice training" OR "psychotherap*" OR "counselling" OR "mental health service*" OR "community health service*" OR "psychosocial intervention*" OR "speech therap*" OR "voice therap*" OR "voice surg*" OR "tracheal cartilage shave" OR "otorhinolaryngolog* surg*" OR "gonadectomy" OR "voice mod*" OR "feminiz*" OR "masculinization" OR "androgenization" OR "penile implantation*" OR "top surgery" ) ) )

## Embase Search Strategy

1. exp gender identity/
2. exp transgender/
3. exp transsexualism/
4. exp cross-dressing/
5. exp gender dysphoria/
6. exp gender reassignment/
7. exp gonadorelin/
8. exp sex hormone/
9. exp estrogen therapy/
10. exp antiestrogen/
11. exp testosterone/
12. exp antiandrogen/
13. exp mastectomy/
14. exp breast reconstruction/
15. exp castration/
16. exp physiotherapy/
17. exp occupational therapy/
18. exp voice training/
19. exp psychotherapy/
20. exp mental health service/
21. exp community care/
22. exp psychosocial intervention/
23. exp speech therapy/
24. exp feminization/
25. exp gonadal disease/
26. exp orchiectomy/
27. exp penile prosthesis implantation/
28. exp hysterectomy/
29. exp hormone substitution/
30. (Gender ident* or transgender* or transperson* or transpeople* or Trans gender* or Transexual* or Trans sexual* or Transman or Transmen or Trans man or Trans men or Transwoman or Transwomen or Trans woman or Trans women or Trans masc* or Transmasc* or Trans fem* or Transfem* or Gender quest* or Gender fluid* or Genderfluid* or Gender non-conforming or Gender nonconforming or Nonbinary or Agender* or Bigender* or Intergender* or Inter-gender* or Multigender or Multi-gender* or Genderqueer or Gender queer or Gender minority or Gender minorities or Gender divers* or Gender incongruent or Gender change or Trans adult* or Trans person* or Trans male* or Trans female* or Cross gender or Crossgender or Transvest* or Cross dress* or two-spirit* or Gender affirm* or gender dysphor* or Gender euphoria or Gender identity disorder* or MTF Patients or Male to female transgender or Female to male transgender or MTF or FTM).mp.
31. (Sex* reassignment* or Gender reassign* or Gender confirm* or Sex* reversal* or Sex* transition* or Sex* change* or Sex* conform* or Sex* affirm* or Genital reconstruct* or Recon* surg* or Cross-sex hormone* or Cross-gender hormone* or Gonadotropin releasing hormone agonist* or GnRH agonist* or Androgen agonist* or Hormon* therap* or Estrogen therap* or Estrogen replace* or Anti-estrogen* or Estrogen antagonist* or Estrogen blocker* or Testosterone therap* or Testosterone replace* or Androgen antagonist* or Anti-androgen* or Androgen blocker* or Mastectom* or Breast reconstruct* or Mammaplast* or Breast endoprosthesis or Breast implant* or Tissue expan* or Thorax surger* or Chest contour* or Chest-wall contour* or Chest reconstruct* or Bottom surg* or Penile reconstruct* or Penile construct* or Testic* construct* or Testicular implant* or Orchiectom* or Vaginoplasty or Vagina* reconstruct* or Vagina* construct* or Penectomy or Metoidioplasty or Phalloplasty or Penile implant* or Genitoplasty or Psych* support* or Psych* care or Physical therap* or Physiotherap* or Occupational therap* or Voice training or Psychotherap* or Counselling or Mental health service* or Community health service* or Psychosocial intervention* or Speech therap* or Voice therap* or Voice surg* or tracheal cartilage shave or otorhinolaryngolog* surg* or Gonadectomy or Voice mod* or Feminiz* or Masculinization or Androgenization or Penile implantation* or Top surgery).mp.
32. (Canad* or British Columbia or Colombie Britannique or Alberta* or Saskatchewan or Manitoba* or Ontario or Quebec or (New Brunswick not New Jersey) or Nouveau Brunswick or Nova Scotia or Nouvelle Ecosse or Prince Edward Island or Newfoundland or Labrador or Nunavut or NWT or Northwest Territories or Yukon or Nunavik or Inuvialuit).mp,jx,in. or (Abbotsford or Airdrie or Ajax or Aurora or Barrie or Belleville or Blainville or Brampton or Brantford or Brossard or Burlington or Burnaby or Caledon or Calgary or Cape Breton or Chatham Kent or Chilliwack or Clarington or Coquitlam or Drummondville or Edmonton or Fredericton or Fort McMurray or Gatineau or Granby or Grande Prairie or Sudbury or Guelph or Halton Hills or Iqaluit or Inuvik or Kamloops or Kawartha Lakes or Kelowna or Kingston or Kitchener or Langley or Laval or Lethbridge or Levis or Longueuil or Maple Ridge or Markham or Medicine Hat or Milton or Mirabel or Mississauga or Moncton or Montreal or Nanaimo or New Westminster or Newmarket or Niagara Falls or Norfolk County or North Bay or North Vancouver or Oakville or Oshawa or Ottawa or Peterborough or Pickering or Port Coquitlam or Prince George or Quebec City or Red Deer or Regina or Repentigny or Richmond or Richmond Hill or Saanich or Saguenay or Saint John or Saint-Hyacinthe or Saint-Jean-sur-Richelieu or Saint-Jerome or Sarnia or Saskatoon or Sault Ste Marie or Sherbrooke or St Albert or St Catharines or St John's or Strathcona County or Surrey or Terrebonne or Thunder Bay or Toronto or Trois-Rivieres or Vancouver or Vaughan or ((Cambridge or (Halifax or Hamilton or London or Victoria or Waterloo or Welland or Whitby or Windsor)) not (UK or Britain or United Kingdom or England or Australia)) or Whitehorse or Winnipeg or Wood Buffalo or Yellowknife).ti,ab,kw,in.
33. ("Concordia University" or "Université du Québec à Chicoutimi" or "Université du Québec à Montréal" or "Université du Québec en Abitibi-Témiscamingue" or "Université du Québec à Trois-Rivières" or "Université du Québec en Outaouais" or "Université du Québec" or "Brock University" or "Concordia Lutheran Theological Seminary" or "Carleton University" or "Dominican University College" or "University of Guelph" or "Lakehead University" or "Laurentian University of Sudbury" or "McMaster University" or "Nipissing University" or "University of Ottawa" or "Saint-Paul University" or "Queen's University").in.
34. ("Memorial University of Newfoundland" or "University of Prince Edward Island" or "Acadia University" or "Acadia Divinity College" or "Atlantic School of Theology" or "Cape Breton University" or "Dalhousie University" or "University of King's College" or "Mount Saint Vincent University" or "Nova Scotia College of Art and Design University" or "Université Sainte-Anne" or "St. Francis Xavier University" or "Saint Mary's University" or "Mount Allison University" or "University of New Brunswick" or "Université de Moncton" or "St. Thomas University" or "Bishop's University" or "McGill University").in.
35. ("Ryerson University" or "University of Toronto" or "St. Augustine's Seminary" or "University of St. Michael's College" or "University of Trinity College" or "Victoria University" or "Knox College" or "Wycliffe College" or "Regis College" or "Trent University" or "University of Waterloo" or "St. Jerome's University" or "Renison University College" or "Conrad Grebel University College" or "University of Western Ontario" or "Brescia University College" or "Huron University College" or "King's College" or "Wilfred Laurier University").in.
36. ("University of Windsor" or "York University" or "Ontario College of Art" or "University of Ontario Institute of Technology" or "Algoma University College" or "University of Sudbury" or "Université de Hearst" or "Huntington University" or "Thorneloe University" or "Brandon University" or "Canadian Mennonite University" or "University of Manitoba" or "Université de Saint-Boniface" or "St. Andrew's College" or "University of Winnipeg" or "University of Regina" or "Campion College" or "Luther College" or "University of Saskatchewan").in.
37. ("College of Emmanuel and St. Chad" or "Lutheran Theological Seminary" or "St. Andrew's College" or "St. Thomas More College" or "Horizon College & Seminary" or "University of Alberta" or "Athabasca University" or "University of Calgary" or "Burman University" or "Concordia University of Edmonton" or "University of Lethbridge" or "The King's University College" or "Ambrose University" or "Grant MacEwan University" or "Mount Royal University" or "University of British Columbia" or "University of Northern British Columbia" or "Royal Roads University" or "Simon Fraser University").in.
38. ("University of Victoria" or "Thompson Rivers University" or "Capilano University" or "Vancouver Island University" or "Emily Carr University of Art and Design" or "Kwantlen Polytechnic University" or "University of the Fraser Valley" or "Yukon University").in.
39. 1 or 2 or 3 or 4 or 5 or 30
40. 6 or 7 or 8 or 9 or 10 or 11 or 12 or 13 or 14 or 15 or 16 or 17 or 18 or 19 or 20 or 21 or 22 or 23 or 24 or 25 or 26 or 27 or 28 or 29 or 31
41. 32 or 33 or 34 or 35 or 36 or 37 or 38
42. 39 and 40 and 41
