# Supplementary material for: Outcome measurement for gender-affirming care in Canada: a systematic review
Source: BMJ Open. 2025 Mar 12;15(3):e091135. doi: 10.1136/bmjopen-2024-091135 (PMC11904330; doi:10.1136/bmjopen-2024-091135)
Supplement: online supplemental file 2 [file bmjopen-15-3-s002.docx]

Supplementary Table 1. Geographic Location and Institution of Included Studies

| **Province of Included Studies** | |
| --- | --- |
| **Province(s)** | **Frequency, n (%)** |
| Alberta | 2 (3.1%) |
| Alberta, British Columbia, Manitoba, Nova Scotia, Ontario, Quebec, Saskatchewan | 1 (1.6%) |
| Atlantic Provinces, Quebec, Ontario, Prairie Provinces, Alberta, British Columbia | 1 (1.6%) |
| British Columbia | 12 (18.8%) |
| Manitoba | 1 (1.6%) |
| Nova Scotia, Quebec, Ontario, Manitoba, Alberta, British Columbia | 1 (1.6%) |
| Ontario | 33 (51.6%) |
| Ontario, British Columbia, Quebec | 1 (1.6%) |
| Quebec | 7 (10.9%) |
| Quebec and Ontario | 1 (1.6%) |
| NR | 4 (6.3%) |
| **City/Cities of Included Studies** | |
| **City/Cities** | **Frequency, n (%)** |
| Vancouver | 10 (15.6%) |
| Burnaby | 1 (1.6%) |
| Edmonton | 2 (3.1%) |
| Halifax, Montreal, Ottawa, Toronto, Hamilton, London, Winnipeg, Calgary, Edmonton, Vancouver | 1 (1.6%) |
| Kingston | 1 (1.6%) |
| Mississauga | 1 (1.6%) |
| Montreal | 7 (10.9%) |
| Montreal, Toronto | 1 (1.6%) |
| Ottawa | 5 (7.8%) |
| Toronto | 24 (37.5%) |
| Toronto, Mississauga | 1 (1.6%) |
| Toronto, Vancouver, Montreal | 1 (1.6%) |
| Winnipeg, Rural Manitoba, First Nation Reserve, Other | 1 (1.6%) |
| NR | 8 (12.5%) |
| **Institutional Affiliation of Included Studies** | |
| **Institution** | **Frequency, n (%)** |
| Brigham and Women’s Hospital | 1 (1.6) |
| Dalhousie University | 1 (1.6%) |
| Doheny Eye Institute, Los Angeles | 1 (1.6%) |
| McMaster University | 1 (1.6%) |
| Queen’s University | 1 (1.6%) |
| University of Alberta | 3 (4.7%) |
| University of British Columbia | 13 (20.3%) |
| University of Manitoba | 1 (1.6%) |
| University of Montreal | 4 (6.3%) |
| University of Ottawa | 5 (7.8%) |
| University of Quebec | 2 (3.1%) |
| University of Sherbrooke | 1 (1.6%) |
| University of Toronto | 27 (42.2%) |
| Western University | 3 (4.7%) |

Supplementary Table 2. Physiologic Parameters Measured

| **Physiologic Outcome** | **Frequency, n (%)** |
| --- | --- |
| 24-hour urinary free-cortisol | 1 (1.2%) |
| Acoustic data (pitch and loudness) | 2 (2.4%) |
| Alanine aminotransferase | 2 (2.4%) |
| Alpha fetoprotein | 1 (1.2%) |
| Androstenedione | 1 (1.2%) |
| Aspartate aminotransferase | 2 (2.4%) |
| Aeta human chorionic gonadotropin | 1 (1.2%) |
| Bone age | 1 (1.2%) |
| Bone mineral density | 1 (1.2%) |
| Creatinine | 2 (2.4%) |
| Dehydroepiandrosterone sulfate | 1 (1.2%) |
| ECG for QTc interval | 1 (1.2%) |
| Estradiol | 6 (7.1%) |
| Free thyroxine | 1 (1.2%) |
| FSH | 4 (4.7%) |
| Genital sensitivity using monofilaments and vulvagesiometer | 1 (1.2%) |
| Glucose | 1 (1.2%) |
| Glycosylated hemoglobin | 2 (2.4%) |
| Growth hormone | 2 (2.4%) |
| Growth parameters | 1 (1.2%) |
| HDL | 1 (1.2%) |
| Hearing test for dichotic stimuli | 1 (1.2%) |
| Hematocrit | 2 (2.4%) |
| Hemoglobin | 2 (2.4%) |
| High-density lipoprotein | 2 (2.4%) |
| IgE level | 1 (1.2%) |
| Insulin | 2 (2.4%) |
| Karyotype | 1 (1.2%) |
| LH | 4 (4.7%) |
| Low-density lipoprotein | 2 (2.4%) |
| Lung function (FEV1) | 1 (1.2%) |
| Oocytes retrieved for preservation | 3 (3.5%) |
| Pathology report for anterior and posterior cervix, endomyometrium, fallopian tubes, and ovaries | 1 (1.2%) |
| Pathology report for granular cell tumour | 1 (1.2%) |
| Pregnancy | 1 (1.2%) |
| Prolactin | 3 (3.5%) |
| PSA | 3 (3.5%) |
| RBC | 2 (2.4%) |
| Serum testosterone | 11 (12.9%) |
| Sex hormone binding globulin | 2 (2.4%) |
| Sputum culture | 1 (1.2%) |
| Total cholesterol | 1 (1.2%) |
| Triglycerides | 2 (2.4%) |
| TSH | 1 (1.2%) |

Supplementary Table 3. Provider-Reported Outcomes Measured

| **Provider-Reported Outcome** | **Frequency, n (%)** |
| --- | --- |
| Cystoscopy | 1 (4%) |
| Lactation induction | 1 (4%) |
| Ophthalmological examination (visual acuity, fundoscopy, optical coherence tomography) | 1 (4%) |
| Postoperative complications | 13 (52%) |
| Provider assessment of patient photographs post-treatment | 3 (12%) |
| Sperm production | 1 (4%) |
| Tanner staging | 1 (4%) |
| Testicular size | 1 (4%) |
| Urine post void residual tests | 1 (4%) |
| Uroflwmetry | 1 (4%) |
| Visual inspection of neovagina | 1 (4%) |

Supplementary Table 4. Patient-Reported Outcomes Measured

| **PROM Administered** | **Specific Concept Measured** | **Construct Measured** | **Number of items** | **Frequency of Use, n (%)** |
| --- | --- | --- | --- | --- |
| ***PROMs measuring gender-related concepts*** | | | | |
| Feminine Gender Identity Scale | History of feminine attitudes and behaviour | Gender-Related Concepts | 37 | 1 (1.5%) |
| Games Inventory | Gender characteristics of games children play | Gender-Related Concepts | 65 | 1 (1.5%) |
| Gender Behaviour Inventory | Masculine or feminine behaviours | Gender-Related Concepts | 48 | 1 (1.5%) |
| Gender Identity Questionnaire for Adolescents (GIQ-Ad) | Gender identity | Gender-Related Concepts | 13 | 2 (3%) |
| Gender Identity/Gender Dysphoria Questionnaire for Adolescents and Adults (GIDYQ) | Gender identity | Gender-Related Concepts | 27 | 2 (3%) |
| Trans Youth CAN Gender Distress Scale | Gender distress | Gender-Related Concepts | 14 | 1 (1.5%) |
| Utrecht Gender Dysphoria Scale | Gender dysphoria | Gender-Related Concepts | 12 | 2 (3%) |
| ***PROMs measuring general health state*** | | | | |
| Canadian Community Health Survey | Health state | General Health State | NR | 1 (1.5%) |
| Canadian Trans Youth Health Survey | General health | General Health State | NR | 1 (1.5%) |
| ***PROMs measuring health-related quality of life*** | | | | |
| EQ-5D | Health-related quality of life | Health-Related Quality of Life | 5 | 1 (1.5%) |
| Migraine Disability Assessment Scores | Impact of headaches on daily life | Health-Related Quality of Life | 7 | 1 (1.5%) |
| ***PROMs measuring healthcare experience*** | | | | |
| Affirm Acceptability Scale | Acceptability of Health Intervention (called AFFIRM) | Healthcare Experience | 17 | 2 (3%) |
| Barriers to Care Scale | Barriers to health care | Healthcare Experience | 12 | 1 (1.5%) |
| Telehealth Usability Questionnaire | Quality of newer-generation virtual care platforms, including usefulness, ease of use, reliability, interface and interaction quality, and satisfaction | Healthcare Experience | 21 | 1 (1.5%) |
| ***PROM measuring outcomes after surgery*** | | | | |
| Satisfaction Questionnaire by Lothstein and Shinar | Readiness for surgery, regrets, satisfaction with appearance of genitals, sexual functioning, clitoral sensitivity | Outcomes after surgery | 59 | 1 (1.5%) |
| ***PROM measuring pain*** | | | | |
| Numeric Pain Rating Scale | Post-surgical pain | Pain | 1 | 1 (1.5%) |
| ***PROM measuring physical health*** | | | | |
| Six-item Physical health component summary score of Short Form Health Survey | Physical health | Physical health | 6 | 1 (1.5%) |
| ***PROMs measuring psychosocial functioning*** | | | | |
| Adult Self-Report | Psychological Functioning | Psychosocial | 126 | 1 (1.5%) |
| Beck Anxiety Inventory | Anxiety | Psychosocial | 21 | 1 (1.5%) |
| Beck Depression Inventory | Depression | Psychosocial | 21 | 6 (9.1%) |
| BODY-Q Chest Module | Satisfaction with appearance of chest and nipple | Psychosocial | 15 | 3 (4.6%) |
| Brief Cope Inventory | Coping after a stressful life event | Psychosocial | 28 | 1 (1.5%) |
| Child Behaviour Checklist | Behavioural problems | Psychosocial | 118 | 1 (1.5%) |
| Cross-Gender Fetishm scale | Erotic arousal with cross-dressing | Psychosocial | 11 | 1 (1.5%) |
| HIV Stigma Scale | HIV-related stigma | Psychosocial | 10 | 1 (1.5%) |
| Hope Scale | Hope | Psychosocial | 12 | 2 (3%) |
| Kessler-6 Scale for Psychological Distress | Psychological distress | Psychosocial | 6 | 1 (1.5%) |
| Modified Depression Scale | Depression | Psychosocial | 6 | 2 (3%) |
| Multidimensional Anxiety Scale for Children (MASC2) | Anxiety | Psychosocial | 50 | 1 (1.5%) |
| Overall Anxiety and Impairment Score | Anxiety | Psychosocial | 5 | 2 (3%) |
| PHQ9 | Depression | Psychosocial | 9 | 1 (1.5%) |
| Piers-Harris Children Self-Concept Scale | Interpersonal competencies | Psychosocial | 60 | 1 (1.5%) |
| Proactive Coping Inventory for Adolescents-A (PCI-A)-Reflective Coping Subscale | Behavioural alternatives | Psychosocial | 11 | 3 (4.6%) |
| Rosenberg Self-Esteem Scale | Self-esteem | Psychosocial | 10 | 1 (1.5%) |
| Satisfaction with Life Scale | Life satisfaction | Psychosocial | 5 | 1 (1.5%) |
| Social Desirability Scale | Tendency to present in a socially desirable manner; | Psychosocial | 33 | 1 (1.5%) |
| Stress Appraisal Measure for Adolescents | Stress | Psychosocial | 14 | 3 (4.6%) |
| Stressors on Families of Trans Youth Checklist | Stress | Psychosocial | 1 | 1 (1.5%) |
| Youth Self-Report | Problem behaviours | Psychosocial | 118 | 1 (1.5%) |
| ***PROM measuring quality of life*** | | | | |
| Flanagan Quality of Life Scale | Satisfaction with interpersonal relationships, material comforts, occupational opportunities | Quality of Life | 15 | 1 (1.5%) |
| ***PROMs measuring sexual functioning*** | | | | |
| Derogatis Sexual Functioning Scale | Sexual functioning | Sexual Functioning | 26 | 1 (1.5%) |
| Erection Hardness Scale | Erection hardness | Sexual Functioning | 1 | 1 (1.5%) |
| Erotic Response and Orientation Scale (EROS) | Sexual orientation and attraction | Sexual Functioning | 16 | 1 (1.5%) |
| Modified Androphilia Scale | Erotic attraction to physically mature males | Sexual Functioning | NR | 1 (1.5%) |
| Modified Gynephilia Scale | Erotic attraction to physically mature females | Sexual Functioning | NR | 1 (1.5%) |
| ***PROM measuring voice function*** | | | | |
| Transsexual Voice Questionnaire | Satisfaction with voice | Voice Function | 30 | 3 (4.6%) |

Supplementary Table 5. CASP Checklist Data (n=52)

| CASP Checklist Item | Rating, n (%) | | | |
| --- | --- | --- | --- | --- |
|  | Yes | No | Can’t Tell | Not Applicable |
| Did the study address a clearly focused issue? | 52 (100) | 0 (0) | 0 (0) | 0 (0) |
| Was the cohort recruited in an acceptable way? | 52 (100) | 0 (0) | 0 (0) | 0 (0) |
| Was the exposure accurately measured to minimise bias? | 50 (96) | 0 (0) | 2 (4) | 0 (0) |
| Was the outcome accurately measured to minimise bias? | 50 (96) | 0 (0) | 2 (4) | 0 (0) |
| Have the authors identified all important confounding factors? | 20 (38) | 30 (58) | 2 (4) | 0 (0) |
| Have they taken account of the confounding factors in the design and/or analysis? | 21 (40) | 29 (56) | 2 (4) | 0 (0) |
| Was the follow up of subjects complete enough? | 40 (77) | 0 (0) | 3 (6) | 9 (17) |
| Was the follow up of subjects long enough? | 40 (77) | 0 (0) | 3 (6) | 9 (17) |
| Do you believe the results? | 49 (94) | 1 (2) | 2 (4) | 0 (0) |
| Can the results be applied to the local population? | 50 (96) | 1 (2) | 1 (2) | 0 (0) |
| Do the results of this study fit with other available evidence? | 50 (96) | 0 (0) | 2 (4) | 0 (0) |

Supplementary Table 6. JBI Checklist Results (n=12)

| JBI Checklist Item | Rating, n (%) | | | |
| --- | --- | --- | --- | --- |
|  | Yes | No | Can’t Tell | Not Applicable |
| Were patient’s demographic characteristics clearly described? | 12 (100) | 0 (0) | 0 (0) | 0 (0) |
| Was the patient’s history clearly described and presented as a timeline? | 12 (100) | 0 (0) | 0 (0) | 0 (0) |
| Was the current clinical condition of the patient on presentation clearly described? | 12 (100) | 0 (0) | 0 (0) | 0 (0) |
| Were diagnostic tests or assessment methods and the results clearly described? | 12 (100) | 0 (0) | 0 (0) | 0 (0) |
| Was the intervention(s) or treatment procedure(s) clearly described? | 12 (100) | 0 (0) | 0 (0) | 0 (0) |
| Was the post-intervention clinical condition clearly described? | 12 (100) | 0 (0) | 0 (0) | 0 (0) |
| Were adverse events (harms) or unanticipated events identified and described? | 9 (75) | 3 (25) | 0 (0) | 0 (0) |
| Does the case report provide takeaway lessons? | 12 (100) | 0 (0) | 0 (0) | 0 (0) |

# Appendix 1: Data Extraction

#### General Information

- **DOI**
- **Title**
- **Year**
- **First Author**
- **First Author Affiliation**
- **Article Type**
- **Study Aim**
- **Study Design**
- **Study Funding**

#### Population Information

- **Recruitment**
- **Inclusion Criteria**
- **Exclusion Criteria**
- **Number of Participants**
- **Gender Identity of Participants**
- **Race/Ethnicity of Participants**
- **Age**
- **Country**/ **Province**/ **City**
- **Gender-Affirming Care Provided**

#### Study Outcomes

- **Health Outcome Measure**
- **Barriers and Enablers to Health Outcome Measurement**
- **Barriers and Enablers to Accessing Gender-Affirming Care**
- **Researcher Reflexivity**
- **Patient and Public Involvement**
- **Data Security**

# Appendix 2: Included Articles

1. Aitken, M., Steensma, T. D., Blanchard, R., VanderLaan, D. P., Wood, H., Fuentes, A., Spegg, C., Wasserman, L., Ames, M., Fitzsimmons, C. L., Leef, J. H., Lishak, V., Reim, E., Takagi, A., Vinik, J., Wreford, J., Cohen-Kettenis, P. T., De Vries, A. L. C., Kreukels, B. P. C., & Zucker, K. J. (2015). Evidence for an Altered Sex Ratio in Clinic-Referred Adolescents with Gender Dysphoria. *The Journal of Sexual Medicine*, *12*(3), 756–763. https://doi.org/10.1111/jsm.12817
2. Anderson, J. A. (2014). Pitch Elevation in Trangendered Patients: Anterior Glottic Web Formation Assisted by Temporary Injection Augmentation. *Journal of Voice*, *28*(6), 816–821. https://doi.org/10.1016/j.jvoice.2014.05.002
3. Armstrong, I., Lacombe-Duncan, A., Shokoohi, M., Persad, Y., Tseng, A., Fung, R., Underhill, A., Côté, P., Machouf, N., Saucier, A., Varriano, B., Brundage, M., Jones, R., Weisdorf, T., Goodhew, J., MacLeod, J., & Loutfy, M. (2023). Feminizing hormone therapy in a Canadian cohort of transgender women with and without HIV. *Antiviral Therapy*, *28*(3), 135965352311825. https://doi.org/10.1177/13596535231182505
4. Bauer, G. R., Lawson, M. L., & Metzger, D. L. (2022). Do Clinical Data from Transgender Adolescents Support the Phenomenon of “Rapid Onset Gender Dysphoria”? *The Journal of Pediatrics*, *243*, 224-227.e2. https://doi.org/10.1016/j.jpeds.2021.11.020
5. Bauer, G. R., Pacaud, D., Couch, R., Metzger, D. L., Gale, L., Gotovac, S., Mokashi, A., Feder, S., Raiche, J., Speechley, K. N., Temple Newhook, J., Ghosh, S., Sansfaçon, A. P., Susset, F., Lawson, M. L., & for the Trans Youth CAN! Research Team. (2021). Transgender Youth Referred to Clinics for Gender-Affirming Medical Care in Canada. *Pediatrics*, *148*(5), e2020047266. https://doi.org/10.1542/peds.2020-047266
6. Blanchard, R., Clemmensen, L. H., & Steiner, B. W. (1985). Social desirability response set and systematic distortion in the self-report of adult male gender patients. *Archives of Sexual Behavior*, *14*(6), 505–516. https://doi.org/10.1007/BF01541751
7. Blanchard, R., Legault, S., & Lindsay, W. R. N. (1987). Vaginoplasty outcome in male-to-female transsexuals. *Journal of Sex & Marital Therapy*, *13*(4), 265–275. https://doi.org/10.1080/00926238708403899
8. Blanchard, R., Steiner, B. W., Clemmensen, L. H., & Dickey, R. (1989). Prediction of Regrets in Postoperative Transsexuals. *The Canadian Journal of Psychiatry*, *34*(1), 43–45. https://doi.org/10.1177/070674378903400111
9. Bonapace-Potvin, M., Lorange, E., Ma, X., Medor, M. C., Bensimon, É., Brassard, P., & Bélanger, M. (2023). The Montréal Classification of urethral lengthening for phalloplasty in transmasculine patients—Surgical techniques and urethral complications. *International Journal of Transgender Health*, *24*(4), 461–468. https://doi.org/10.1080/26895269.2022.2104418
10. Bonapace-Potvin, M., Pepin, M., Navals, P., Medor, M. C., Lorange, E., & Bensimon, É. (2023). Facial Gender-Affirming Surgery: Frontal Bossing Surgical Techniques, Outcomes and Safety. *Aesthetic Plastic Surgery*, *47*(4), 1353–1361. https://doi.org/10.1007/s00266-022-03180-3
11. Chen, I., Nguyen, V., Hodge, M., Mallick, R., Gagné, H., Singh, S. S., Choudhry, A. J., Xie, R., Liao, Y., & Wen, S.-W. (2020). Surgical Outcomes for Transgender Men Undergoing Hysterectomy. *Journal of Obstetrics and Gynaecology Canada*, *42*(1), 25–30. https://doi.org/10.1016/j.jogc.2019.05.009
12. Chiniara, L. N., Bonifacio, H. J., & Palmert, M. R. (2018). Characteristics of Adolescents Referred to a Gender Clinic: Are Youth Seen Now Different from Those in Initial Reports? *Hormone Research in Paediatrics*, *89*(6), 434–441. https://doi.org/10.1159/000489608
13. Cho, K., Harjee, R., Roberts, J., & Dunne, C. (2020). Fertility preservation in a transgender man without prolonged discontinuation of testosterone: A case report and literature review. *F&S Reports*, *1*(1), 43–47. https://doi.org/10.1016/j.xfre.2020.03.003
14. Clark, B. A., Veale, J. F., Townsend, M., Frohard-Dourlent, H., & Saewyc, E. (2018). Non-binary youth: Access to gender-affirming primary health care. *International Journal of Transgenderism*, *19*(2), 158–169. https://doi.org/10.1080/15532739.2017.1394954
15. Cohen, H., & Forget, H. (1995). Auditory Cerebral Lateralization Following Cross-Gender Hormone Therapy. *Cortex*, *31*(3), 565–573. https://doi.org/10.1016/S0010-9452(13)80067-6
16. Craig, S. L., & Austin, A. (2016). The AFFIRM open pilot feasibility study: A brief affirmative cognitive behavioral coping skills group intervention for sexual and gender minority youth. *Children and Youth Services Review*, *64*, 136–144. https://doi.org/10.1016/j.childyouth.2016.02.022
17. Craig, S. L., Eaton, A. D., Leung, V. W. Y., Iacono, G., Pang, N., Dillon, F., Austin, A., Pascoe, R., & Dobinson, C. (2021). Efficacy of affirmative cognitive behavioural group therapy for sexual and gender minority adolescents and young adults in community settings in Ontario, Canada. *BMC Psychology*, *9*(1), 94. https://doi.org/10.1186/s40359-021-00595-6
18. Craig, S. L., Leung, V. W. Y., Pascoe, R., Pang, N., Iacono, G., Austin, A., & Dillon, F. (2021). AFFIRM Online: Utilising an Affirmative Cognitive–Behavioural Digital Intervention to Improve Mental Health, Access, and Engagement among LGBTQA+ Youth and Young Adults. *International Journal of Environmental Research and Public Health*, *18*(4), 1541. https://doi.org/10.3390/ijerph18041541
19. Davies, S. M., & Johnston, J. R. (2015). Exploring the validity of the transsexual voice questionnaire for male-to-female transsexuals. *Canadian Journal of Speech-Language Pathology and Audiology*, *39*, 40–51.
20. El-Hadi, H., Stone, J., Temple-Oberle, C., & Harrop, A. R. (2018). Gender-Affirming Surgery for Transgender Individuals: Perceived Satisfaction and Barriers to Care. *Plastic Surgery*, *26*(4), 263–268. https://doi.org/10.1177/2292550318767437
21. Fung, R., Greenaway, M. K., & McEvenue, G. (2021). Gynecomastia in a Transgender Boy: A Case Report. *AACE Clinical Case Reports*, *7*(6), 350–352. https://doi.org/10.1016/j.aace.2021.05.003
22. Fung, R., Hellstern-Layefsky, M., & Lega, I. (2017). Is a lower dose of cyproterone acetate as effective at testosterone suppression in transgender women as higher doses? *International Journal of Transgenderism*, *18*(2), 123–128. https://doi.org/10.1080/15532739.2017.1290566
23. Fung, R., Hellstern-Layefsky, M., Tastenhoye, C., Lega, I., & Steele, L. (2016). Differential Effects of Cyproterone Acetate vs Spironolactone on Serum High-Density Lipoprotein and Prolactin Concentrations in the Hormonal Treatment of Transgender Women. *The Journal of Sexual Medicine*, *13*(11), 1765–1772. https://doi.org/10.1016/j.jsxm.2016.09.012
24. Gale, J., Magee, B., Forsyth-Greig, A., Visram, H., & Jackson, A. (2021). Oocyte cryopreservation in a transgender man on long-term testosterone therapy: A case report. *F&S Reports*, *2*(2), 249–251. https://doi.org/10.1016/j.xfre.2021.02.006
25. Hallarn, J., Bauer, G. R., Potter, E., Wilcox, H., Newfeld, J., Krakowsky, Y., Ravel, J., & Prodger, J. L. (2023). Gynecological concerns and vaginal practices and exposures among transfeminine individuals who have undergone vaginoplasty. *The Journal of Sexual Medicine*, *20*(11), 1344–1352. https://doi.org/10.1093/jsxmed/qdad109
26. Hana, T., Raveendran, L., Grober, E., Potter, E., Blodgett, N., & Krakowsky, Y. (2020). Initial clinical experience with simple orchiectomy procedures in the context of transition-related surgeries. *International Journal of Transgender Health*, *21*(4), 403–409. https://doi.org/10.1080/26895269.2020.1774030
27. Hardy, T. L. D., Rieger, J. M., Wells, K., & Boliek, C. A. (2020). Acoustic Predictors of Gender Attribution, Masculinity–Femininity, and Vocal Naturalness Ratings Amongst Transgender and Cisgender Speakers. *Journal of Voice*, *34*(2), 300.e11-300.e26. https://doi.org/10.1016/j.jvoice.2018.10.002
28. Heard, J., Morris, A., Kirouac, N., Ducharme, J., Trepel, S., & Wicklow, B. (2018). Gender dysphoria assessment and action for youth: Review of health care services and experiences of trans youth in Manitoba. *Paediatrics & Child Health*, *23*(3), 179–184. https://doi.org/10.1093/pch/pxx156
29. Jones, C. A., Reiter, L., & Greenblatt, E. (2016). Fertility preservation in transgender patients. *International Journal of Transgenderism*, *17*(2), 76–82. https://doi.org/10.1080/15532739.2016.1153992
30. Jung, H., Chen, M. L., Wassersug, R., Mukherjee, S., Kumar, S., Mankowski, P., Genoway, K., & Kavanagh, A. (2023). Urethroplasty Outcomes for Pars Fixa Urethral Strictures Following Gender-affirming Phalloplasty and Metoidioplasty: A Retrospective Study. *Urology*, *182*, 89–94. https://doi.org/10.1016/j.urology.2023.07.009
31. Kaur, M. N., Gallo, L., Wang, Y., Rae, C., McEvenue, G., Semple, J., Johnson, N., Savard, K., Pusic, A. L., Coon, D., & Klassen, A. F. (2023). Health state utility values in patients undergoing chest masculinization surgery. *Journal of Plastic, Reconstructive & Aesthetic Surgery*, *81*, 26–33. https://doi.org/10.1016/j.bjps.2023.02.004
32. Khatchadourian, K., Amed, S., & Metzger, D. L. (2014). Clinical Management of Youth with Gender Dysphoria in Vancouver. *The Journal of Pediatrics*, *164*(4), 906–911. https://doi.org/10.1016/j.jpeds.2013.10.068
33. Khorrami, A., Kumar, S., Bertin, E., Wassersug, R., O’Dwyer, C., Mukherjee, S., Witherspoon, L., Mankowski, P., Genoway, K., & Kavanagh, A. G. (2022). The Sexual Goals of Metoidioplasty Patients and Their Attitudes Toward Using PDE5 Inhibitors and Intracavernosal Injections as Erectile Aids. *Sexual Medicine*, *10*(3), 1–8. https://doi.org/10.1016/j.esxm.2022.100505
34. Klassen, A. F., Kaur, M., Poulsen, L., Fielding, C., Geerards, D., Van De Grift, T. C., Hoogbergen, M., Juhl, C. B., Lorenzen, M. M., McEvenue, G., McLean, H., Moliver, C., Mullender, M. G., Panchapakesan, V., Repo, J. P., Rose, M., Sørensen, J. A., Støving, R. K., & Pusic, A. L. (2018). Development of the BODY-Q Chest Module Evaluating Outcomes following Chest Contouring Surgery. *Plastic & Reconstructive Surgery*, *142*(6), 1600–1608. https://doi.org/10.1097/PRS.0000000000004978
35. Knox, A. D. C., Ho, A. L., Leung, L., Hynes, S., Tashakkor, A. Y., Park, Y. S., Macadam, S. A., & Bowman, C. C. (2017). A Review of 101 Consecutive Subcutaneous Mastectomies and Male Chest Contouring Using the Concentric Circular and Free Nipple Graft Techniques in Female-to-Male Transgender Patients. *Plastic & Reconstructive Surgery*, *139*(6), 1260e–1272e. https://doi.org/10.1097/PRS.0000000000003388
36. Kogachi, K., Konstas, A., Karanjia, R., & Sadun, A. A. (n.d.). *Endovascular Stenting in a Transgender Patient With Idiopathic Intracranial Hypertension*.
37. Kumar, S., Bertin, E., O’Dwyer, C., Khorrami, A., Wassersug, R., Mukherjee, S., Mehra, N., Dahl, M., Genoway, K., & Kavanagh, A. G. (2023). Serum estradiol levels decrease after oophorectomy in transmasculine individuals on testosterone therapy. *Asian Journal of Andrology*, *25*(3), 309–313. https://doi.org/10.4103/aja202262
38. Kumar, S., Tyldesley, S., Poon, C. I., Saunders, J. T. W., & Hoag, C. C. (2023). Case – Laparoscopic radical prostatectomy in a transgender woman after gender-affirming vaginoplasty. *Canadian Urological Association Journal*, *18*(3). https://doi.org/10.5489/cuaj.8387
39. Lacombe-Duncan, A., Newman, P. A., Bauer, G. R., Logie, C. H., Persad, Y., Shokoohi, M., O’Brien, N., Kaida, A., De Pokomandy, A., & Loutfy, M. (2019). Gender-affirming healthcare experiences and medical transition among transgender women living with HIV: A mixed-methods study. *Sexual Health*, *16*(4), 367. https://doi.org/10.1071/SH19011
40. Lam, G. Y., Goodwin, J., Wilcox, P., & Quon, B. S. (2020). Worsening pulmonary outcomes during sex reassignment therapy in a transgender female with cystic fibrosis (CF) and asthma/allergic bronchopulmonary aspergillosis: A case report. *BMC Pulmonary Medicine*, *20*(1), 234. https://doi.org/10.1186/s12890-020-01272-x
41. LeBreton, M., Courtois, F., Journel, N. M., Beaulieu-Prévost, D., Bélanger, M., Ruffion, A., & Terrier, J.-É. (2017). Genital Sensory Detection Thresholds and Patient Satisfaction with Vaginoplasty in Male-to-Female Transgender Women. *The Journal of Sexual Medicine*, *14*(2), 274–281. https://doi.org/10.1016/j.jsxm.2016.12.005
42. Lindsay, W. R. (1979). Creation of a male chest in female transsexuals. *Annals of Plastic Surgery*, *3*(1), 39–46.
43. McEvenue, G., Xu, F. Z., Cai, R., & McLean, H. (2018). Female-to-Male Gender Affirming Top Surgery: A Single Surgeon’s 15-Year Retrospective Review and Treatment Algorithm. *Aesthetic Surgery Journal*, *38*(1), 49–57. https://doi.org/10.1093/asj/sjx116
44. Miller, N., Bédard, Y. C., Cooter, N. B., & Shaul, D. L. (1986). Histological changes in the genital tract in transsexual women following androgen therapy. *Histopathology*, *10*(7), 661–669. https://doi.org/10.1111/j.1365-2559.1986.tb02520.x
45. Navabi, B., Tang, K., Khatchadourian, K., & Lawson, M. L. (2021). Pubertal Suppression, Bone Mass, and Body Composition in Youth With Gender Dysphoria. *Pediatrics*, *148*(4), e2020039339. https://doi.org/10.1542/peds.2020-039339
46. Nayman, T., Hébert, M., & Ospina, L. H. (2021). Idiopathic intracranial hypertension in a pediatric transgender patient. *American Journal of Ophthalmology Case Reports*, *24*, 101208. https://doi.org/10.1016/j.ajoc.2021.101208
47. Oberc, A., Armstrong, K., Ko, H.-M., Grant, A., Mullen, J. B. M., & Williams, P. (2022). Case report of a breast granular cell tumor in a young transgender man. *International Journal of Surgery Case Reports*, *93*, 106978. https://doi.org/10.1016/j.ijscr.2022.106978
48. Obiezu, C. V., Giltay, E. J., Magklara, A., Scorilas, A., Gooren, L. J. G., Yu, H., Howarth, D. J. C., & Diamandis, E. P. (2000). Serum and Urinary Prostate-specific Antigen and Urinary Human Glandular Kallikrein Concentrations Are Significantly Increased after Testosterone Administration in Female-to-Male Transsexuals. *Clinical Chemistry*, *46*(6), 859–862. https://doi.org/10.1093/clinchem/46.6.859
49. Obiezu, C. V., Giltay, E. J., Magklara, A., Scorilas, A., Gooren, L., Yu, H., & Diamandis, E. P. (n.d.). *DRAMATIC SUPPRESSION OF PLASMA AND URINARY PROSTATE SPECIFIC ANTIGEN AND HUMAN GLANDULAR KALLIKREIN BY ANTIANDROGENS IN MALE-TO-FEMALE TRANSSEXUALS*.
50. Potter, E., Sivagurunathan, M., Armstrong, K., Barker, L. C., Du Mont, J., Lorello, G. R., Millman, A., Urbach, D. R., & Krakowsky, Y. (2023). Patient reported symptoms and adverse outcomes seen in Canada’s first vaginoplasty postoperative care clinic. *Neurourology and Urodynamics*, *42*(2), 523–529. https://doi.org/10.1002/nau.25132
51. Prior, J. C., Vigna, Y. M., & Watson, D. (1989). Spironolactone with physiological female steroids for presurgical therapy of male-to-female transsexualism. *Archives of Sexual Behavior*, *18*(1), 49–57. https://doi.org/10.1007/BF01579291
52. Pullen Sansfaçon, A., Temple-Newhook, J., Suerich-Gulick, F., Feder, S., Lawson, M. L., Ducharme, J., Ghosh, S., Holmes, C., & On behalf of the Stories of Gender-Affirming Care Team. (2019). The experiences of gender diverse and trans children and youth considering and initiating medical interventions in Canadian gender-affirming speciality clinics. *International Journal of Transgenderism*, *20*(4), 371–387. https://doi.org/10.1080/15532739.2019.1652129
53. Silva, C., Fung, A., Irvine, M. A., Ziabakhsh, S., & Hursh, B. E. (2021). Usability of Virtual Visits for the Routine Clinical Care of Trans Youth during the COVID-19 Pandemic: Youth and Caregiver Perspectives. *International Journal of Environmental Research and Public Health*, *18*(21), 11321. https://doi.org/10.3390/ijerph182111321
54. Stein, M. J., Grigor, E., Hardy, J., & Jarmuske, M. (2021). Surgical and patient-reported outcomes following double incision and free nipple grafting for female to male gender affirmation: Does obesity make a difference? *Journal of Plastic, Reconstructive & Aesthetic Surgery*, *74*(8), 1743–1751. https://doi.org/10.1016/j.bjps.2020.12.004
55. Taillefer, V., Kelley, J., Marsolais, S., Chiniara, L., & Chadi, N. (2023). Expected vs. Perceived effects of gender-affirming hormone therapy among transmasculine adolescents. *Journal of Pediatric Endocrinology and Metabolism*, *36*(11), 1072–1078. https://doi.org/10.1515/jpem-2023-0119
56. Todd, C. M., Yu, A., Lay, C., & Lagman-Bartolome, A. M. (2023). Effect of testosterone therapy on migraine frequency and disability in two transgender patients: A case report. *BMJ Case Reports*, *16*(1), e251895. https://doi.org/10.1136/bcr-2022-251895
57. Waldner, R. C., Doulla, M., Atallah, J., Rathwell, S., & Grimbly, C. (2023). Leuprolide Acetate and QTc Interval in Gender-Diverse Youth. *Transgender Health*, *8*(1), 84–88. https://doi.org/10.1089/trgh.2021.0102
58. Wamboldt, R., Shuster, S., & Sidhu, B. S. (2021). Lactation Induction in a Transgender Woman Wanting to Breastfeed: Case Report. *The Journal of Clinical Endocrinology & Metabolism*, *106*(5), e2047–e2052. https://doi.org/10.1210/clinem/dgaa976
59. Wassersug, R., Gray, R. E., Barbara, A., Trosztmer, C., Raj, R., & Sinding, C. (2007). Experiences of Transwomen with Hormone Therapy. *Sexualities*, *10*(1), 101–122. https://doi.org/10.1177/1363460707072957
60. Watt, S. O., Tskhay, K. O., & Rule, N. O. (2018). Masculine Voices Predict Well-Being in Female-to-Male Transgender Individuals. *Archives of Sexual Behavior*, *47*(4), 963–972. https://doi.org/10.1007/s10508-017-1095-1
61. White, J., Jackson, A., Druce, I., & Gale, J. (2024). Oocyte cryopreservation and reciprocal in vitro fertilization in a transgender man on long term testosterone gender-affirming hormone therapy: A case report. *F&S Reports*, *5*(1), 111–113. https://doi.org/10.1016/j.xfre.2023.11.004
62. Wilson, D. M., Kiang, T. K. L., & Ensom, M. H. H. (2018). Pharmacokinetics, safety, and patient acceptability of subcutaneous versus intramuscular testosterone injection for gender-affirming therapy: A pilot study. *American Journal of Health-System Pharmacy*, *75*(6), 351–358. https://doi.org/10.2146/ajhp170160
63. Zucker, K. J., Bradley, S. J., Doering, R. W., & Lozinski, J. A. (1985). Sex-typed behavior in cross-gender-identified children: Stability and change at a one-year follow-up. *Journal of the American Academy of Child Psychiatry*, *24*(6), 710–719. https://doi.org/10.1016/s0002-7138(10)60114-8
64. Zucker, K. J., Bradley, S. J., Owen-Anderson, A., Singh, D., Blanchard, R., & Bain, J. (2010). Puberty-Blocking Hormonal Therapy for Adolescents with Gender Identity Disorder: A Descriptive Clinical Study. *Journal of Gay & Lesbian Mental Health*, *15*(1), 58–82. https://doi.org/10.1080/19359705.2011.530574
