# Supplementary material for: Outcome measurement for gender-affirming care in Canada: a systematic review
Source: BMJ Open. 2025 Mar 12;15(3):e091135. doi: 10.1136/bmjopen-2024-091135 (PMC11904330; doi:10.1136/bmjopen-2024-091135)
Supplement: Supplementary file 3 [file bmjopen-2024-091135.reviewer_comments.pdf]

PEER REVIEW HISTORY

BMJ Open publishes all reviews undertaken for accepted manuscripts. Reviewers are asked to complete a checklist review form and are provided with free text boxes to elaborate on their assessment. These free text comments are reproduced below.

ARTICLE DETAILS

Title (Provisional)

Outcome Measurement for Gender-Affirming Care in Canada: A Systematic Review

Authors

Jackman, Liam; Chan, Cynthia; Garvilles, Micon; Kamran, Rakhshan

VERSION 1 - REVIEW

**Reviewer**	**1**
**Name**	**Thorne, Nat**
**Affiliation**	**Nottingham Centre for Transgender Health , Nottingham Centre for Transgender Health**
**Date**	**20-Aug-2024**
**COI**	**None**

This study provides details of the current state of outcome measurements for gender-affirming care in Canada. This paper also looks at barriers to accessing gender affirming care.

This paper provides important information and fills a gap in our knowledge in this area. The authors have provided a comprehensive paper which is important in this area of research. The paper is also very well written with clear understandable language.

The corrections I would like to suggest are…

Page three, line 19. Consider moving 'for gender-affirming care in Canada' to the end of the sentence so we know all of the analysis only applies to Canada.

Page three, line 29. Maybe consider using the word 'and' between 'perspectives' and 'failing' instead of a comma.

Page four, line 29. May be consider specifying gender affirming care rather than just saying 'care'

Page six, line 17. A citation for this increase in referrals would be useful here.

Page seven, line 37. Where were the patients or public partners sourced from? Might be worth adding that here.

Page eight, line 37. So far, the paper does not specify if the patients are young people or adults. The assumption is that you are discussing adult services only, but might be worth clarifying this either in the introduction or here. Later it does become clear it is all ages, but mention that early on.

Page 20, line 24. Individual should be plural at the start of this sentence.

Page 20, line 38. Should 'administrate' be 'administration'?

Page 24, line one. Maybe add a discussion about how samples were very white and this is either the result of poor demographic collection or a wider issue in regards to the disparities and barriers to care for black and brown patients.

Reviewer 2

Name Kearns, Seán

Affiliation University College Dublin, School of Nursing, Midwifery and Health Sciences

Date 10-Jan-2025

COI None

Structured Feedback for the Authors

Overall, I enjoyed this paper and I think it is an incredibly important area of GAC. I like how the author has sub-divided outcome measures. It was clear and easy to understand. The scope is limited to Canada but that is justified by the reviewers.

I have a few suggestions that I think would strengthen the paper overall. Best of luck.

1. Scope of the Review

• Observation: The scope of this review aligns more closely with that of a scoping review rather than a systematic review. I think you scoped the literature well in a systematic manner, but I am not convinced that this reaches the criteria of a systematic review.

• Feedback: Consider reframing the study as a scoping review. The broad objectives, inclusion of heterogeneous studies, lack of meta-analysis, and focus on identifying gaps are characteristic of a scoping review. This reframing would better align the methodology and findings with the review's goals and expectations.

2. Patient and Public Involvement (PPI)

• Observation: The review mentions PPI (Page 8) but lacks sufficient detail about how engagement was conducted.

• Feedback: Authentic PPI requires clear and transparent reporting of how patients or the public were involved, including their role in the research design, execution, and analysis. Without this detail, the claim of PPI lacks credibility. Please provide explicit information about the methods, scope, and impact of PPI activities. If PPI was not meaningfully conducted, consider omitting this claim.

3. Reflexivity and Positionality

• Observation: The review criticizes the lack of reflexivity and positionality across studies but fails to address this gap within its own work.

• Feedback: Including reflexivity and positionality statements is important, particularly when researching marginalized populations. Discuss the authors' own biases, assumptions, and positionality, as well as how these factors may have influenced study design, interpretation, and conclusions.

4. Visual Representation of Data

• Observation: While the tables are comprehensive, they could be enhanced with visual elements like graphs and pie charts to increase the impact and accessibility of findings.

• Feedback: Consider converting key tables into visual representations to improve readability and engagement. For example, pie charts or bar graphs could depict the distribution of study locations, types of outcomes measured, or demographic breakdowns. This will make the data more compelling and easier to interpret.

5. Feedback on Quality Assessment

• Observation: While the study utilizes established tools like the CASP, Oxford level of evidence and JBI checklists for quality assessment, there is insufficient narrative explanation about the scoring process and its implications. The lack of detailed interpretation limits the reader's understanding of how quality influenced the synthesis and conclusions.

• Feedback: Recommend including more narrative text to explain:

o Scoring Process: Clarify how the quality assessment scores were assigned, who conducted the assessments, and whether assessments were conducted independently or collaboratively.

o Implications of Scores: Provide more insight into how the quality scores impacted the inclusion of studies or the weighting of their findings in the analysis. Or did you include them all and if so why?

o Author Commentary: Include a brief discussion of the authors' opinions on the overall quality of the included studies and the implications for the robustness of the review's findings. For example, what does the observed variability in study quality indicate about the current state of research on gender-affirming care?

6. Discussion and Policy Implications

• Observation: While recommendations are made as to how this could impact policy in Canada and healthcare, the feasibility and implementation pathways are not discussed in depth.

• Feedback: Strengthen the discussion by outlining practical steps, resources, and collaborations needed to implement the recommendations. For example:

o What institutional, financial, or systemic changes are required?

o Which stakeholders should be involved in the process?

o Are there existing frameworks or pilot programs that can serve as models? You mentioned the UK's model, could you expand more?

7. Feedback on Word Limit and Depth of Discussion

• Observation: At 2,500 words, the document is relatively short for a review paper tackling such a complex topic as gender-affirming care. This brevity limits the depth of analysis, particularly in the discussion section, where a more critical and analytical approach is needed.

• Feedback: Recommend an expansion of the discussion section to provide a deeper, more analytical interpretation of the findings. I would meet as a research team again and think about why are PROM's so important and in particular in this area? What considerations does that have for clinicians, for patients, for activism?

VERSION 1 - AUTHOR RESPONSE

Comment	Response	Change to the Manuscript
**Reviewer 1**		
This study provides details of the current state of outcome measurements for gender-affirming care in Canada. This paper also looks at barriers to accessing gender affirming care.<br><br>This paper provides important information and fills a gap in our knowledge in this area. The authors have provided a comprehensive paper which is important in this area of research. The paper is also very well	Thank you for this comment, we appreciate the time you took to review our article and appreciate your helpful comments to improve our paper.	N/A

written with clear understandable language.		
Page three, line 19. Consider moving ‘for gender-affirming care in Canada’ to the end of the sentence so we know all of the analysis only applies to Canada.	Thank you for bringing this to our attention. This section of the paper has been removed per Editor comments as it does not meet BMJ Open formatting requirements.	Section removed to ensure paper fits BMJ Open formatting requirements.
Page three, line 29. Maybe consider using the word ‘and’ between ‘perspectives’ and ‘failing’ instead of a comma.	Thank you for bringing this to our attention. This section of the paper has been removed per Editor comments as it does not meet BMJ Open formatting requirements.	Section removed to ensure paper fits BMJ Open formatting requirements.
Page four, line 29. May be consider specifying gender affirming care rather than just saying ‘care’	Thank you for this comment, we agree and have included this change.	Specified care as “gender-affirming care”:<br><br>Inclusion criteria were original articles involving transgender or gender-diverse (TGD) patients receiving gender-affirming care in Canada.
Page six, line 17. A citation for this increase in referrals would be useful here.	Thank you for this comment, we have clarified the reference here.	Reference included:<br><br>Canada has seen a tenfold increase in referrals to GAC over the last 10 years[4], reflecting an emerging acceptance and need for GAC.
Page seven, line 37. Where were the patients or public partners sourced from? Might be worth adding that here.		
Page eight, line 37. So far, the paper does not specify if the patients are young people or adults. The assumption is that you are discussing adult services only, but might be worth clarifying this either in the introduction or here. Later it does become clear it is all ages, but mention that early on.	Thank you for this comment, we agree that this section can be strengthened with more detail. We have included more detail in this revision.	Increased information on PPI included:<br><br>This study includes patient and public involvement (PPI). Seven members representing the transgender and gender diverse community ranging from young adults to adults were recruited through contacting local charities (i.e., Gender Identity Research and Education Society), online community transgender support groups, and recruitment of one individual from an existing patient group with a separate research team.

		PPI were involved in providing feedback on the research question (i.e., supporting the importance of reviewing outcome measurement in Canadian gender-affirming care). PPI meetings were held online over Microsoft Teams, where a meeting agenda was circulated prior to online meetings. An average of 2-3 meetings were held per year with regular email communication in the interim. During meetings, disagreements between PPI were resolved through discussion until all participants agreed on steps forward. PPI aided in identifying search terms, providing feedback on the relevance and importance of study findings, and providing perspective based on personal experiences.
Page 20, line 24. Individual should be plural at the start of this sentence.	Thank you for bringing this to our attention, we have corrected this.	Correction of sentence to pluralize "individual":<br><br>"Individuals also report that provincial health insurance covered some…"
Page 20, line 38. Should 'administrate' be 'administration'?	Thank you for bringing this to our attention, we have corrected this.	Correction of sentence:<br><br>"…documentation and administration processes,…"
Page 24, line one. Maybe add a discussion about how samples were very white and this is either the result of poor demographic collection or a wider issue in regards to the disparities and barriers to care for black and brown patients.	Thank you for this comment. We agree that this is an important point to discuss and have included additional discussion around this.	Additional discussion added on racial disparities for care:<br><br>"The studies included in this systematic review also predominantly focused on participants identified as White, highlighting a limitation in the current research landscape regarding gender-affirming care in Canada. This demographic homogeneity raises concerns about representativeness of study populations and points to potential issues in data collection practices, where demographic variables such as race and ethnicity may not

		have been adequately captured. Alternatively, this skewed representation may reflect broader disparities in access to gender-affirming care, suggesting that Black and Brown patients face significant barriers to receiving care or participating in research studies. The lack of racial diversity in the study may mask critical differences in outcomes and experiences across diverse populations. Black and Brown transgender and gender-diverse individuals often encounter unique challenges, including systemic discrimination, socioeconomic inequities, and reduced access to healthcare services, all of which can influence both their healthcare experiences and outcomes. Therefore, the overrepresentation of White participants in the reviewed studies limits the generalizability of the findings and underscores the urgent need for more inclusive research practices. Addressing these disparities requires targeted efforts to improve demographic data collection, ensure equity in research participation, and enhance access to gender-affirming care for all patients, irrespective of racial or ethnic background.”
**Reviewer 2**		
Overall, I enjoyed this paper and I think it is an incredibly important area of GAC. I like how the author has sub-divided outcome measures. It was clear and easy to understand. The scope is limited to Canada but that is justified by the reviewers.<br><br>I have a few suggestions that I think would	Thank you for this comment and your time in reviewing our paper. We appreciate your helpful comments and have addressed your feedback in this revision.	N/A

strengthen the paper overall. Best of luck.		
1. Scope of the Review<br>• Observation: The scope of this review aligns more closely with that of a scoping review rather than a systematic review. I think you scoped the literature well in a systematic manner, but I am not convinced that this reaches the criteria of a systematic review.<br>• Feedback: Consider reframing the study as a scoping review. The broad objectives, inclusion of heterogeneous studies, lack of meta-analysis, and focus on identifying gaps are characteristic of a scoping review. This reframing would better align the methodology and findings with the review's goals and expectations.	Thank you for this comment, this is an important point for discussion. This paper is a systematic review without meta-analysis, which is a distinct type of systematic review. While this review did not include a meta-analysis due to the heterogeneity of included studies, it adhered to established systematic review methodologies, including a predefined protocol, quality assessment, and appraisal of included studies. The objective was not to map the existing literature (as in scoping reviews) but to evaluate the quality and consistency of outcome measurement in gender-affirming care. Given these methodological rigor and objectives, the study remains aligned with the criteria of a systematic review rather than a scoping review. However, we acknowledge the heterogeneity of included studies and the absence of meta-analysis as limitations that impact the synthesis of quantitative findings.	Additional limitations included in the article to discuss the limitations of this systematic review without meta-analysis in line with points raised by reviewer.<br><br>"Further, due to the heterogeneity of included articles, a meta-analysis was unable to be performed, and a narrative synthesis was conducted."
2. Patient and Public Involvement (PPI)<br>• Observation: The review mentions PPI (Page 8) but lacks sufficient detail about how engagement was conducted.<br>• Feedback: Authentic PPI requires clear and transparent reporting of how patients or the public were involved, including their role in the research design, execution, and analysis. Without this detail, the claim of PPI lacks credibility. Please	Thank you for this comment. We agree and have included additional discussion on PPI.	Increased information on PPI included:<br><br>This study includes patient and public involvement (PPI). Seven members representing the transgender and gender diverse community ranging from young adults to adults were recruited through contacting local charities (i.e., Gender Identity Research and Education Society), online community transgender support groups, and recruitment of one individual from an existing patient group

provide explicit information about the methods, scope, and impact of PPI activities. If PPI was not meaningfully conducted, consider omitting this claim.		with a separate research team. PPI were involved in providing feedback on the research question (i.e., supporting the importance of reviewing outcome measurement in Canadian gender-affirming care). PPI meetings were held online over Microsoft Teams, where a meeting agenda was circulated prior to online meetings. An average of 2-3 meetings were held per year with regular email communication in the interim. During meetings, disagreements between PPI were resolved through discussion until all participants agreed on steps forward. PPI aided in identifying search terms, providing feedback on the relevance and importance of study findings, and providing perspective based on personal experiences.
3. Reflexivity and Positionality<br>• Observation: The review criticizes the lack of reflexivity and positionality across studies but fails to address this gap within its own work.<br>• Feedback: Including reflexivity and positionality statements is important, particularly when researching marginalized populations. Discuss the authors' own biases, assumptions, and positionality, as well as how these factors may have influenced study design, interpretation, and conclusions.	Thank you for bringing this to our attention. We agree and have included reflexivity and positionality statements to our study.	Inclusion of reflexivity and positionality statement:<br><br>"This review was conducted by a multidisciplinary team with diverse expertise in research methodology, systematic reviews, gender-affirming care, and statistical analysis. The senior author (MD, DPhil) has a background in systematic review methodology and completed a doctoral dissertation at the University of Oxford focused on outcome measurement in gender-affirming care. As a member of the LGBTQ+ community, they bring both lived experience and academic expertise to this work. Another team member (MD candidate, PharmD, MSc) is also a member of the LGBTQ+ community and has extensive experience conducting systematic reviews and research on gender-affirming care. A third team member (MD) is a clinician

		researcher who provides gender-affirming care in a clinical setting and has a research background in this area, contributing valuable practical insights. The final author (DPhil candidate) has expertise in research methodology, statistics, and review articles, ensuring a rigorous analytic approach. We acknowledge that our collective identities, professional experiences, and advocacy roles may shape our perspectives on gender-affirming care and outcome measurement. While this expertise strengthens our ability to critically evaluate the literature, it also introduces potential biases, such as a heightened awareness of structural barriers in healthcare and a commitment to advancing equitable and inclusive research. To mitigate these biases, we adhered to established systematic review methodologies, including predefined inclusion criteria, transparent data extraction, and critical appraisal of study quality. We also engaged in ongoing reflexive discussions throughout the review process to ensure that interpretations were grounded in the data rather than personal assumptions. Furthermore, we recognize the limitations of existing research in this field, particularly the lack of racial and ethnic diversity in study samples. While this issue is highlighted in our discussion, we acknowledge that our own positionality as researchers with access to academic and medical institutions may influence how we perceive and frame these disparities.”
4. Visual Representation of Data<br>• Observation: While the	Thank you for this comment, this is a good	New figures included for: province of included studies (Figure 2), cities of included

tables are comprehensive, they could be enhanced with visual elements like graphs and pie charts to increase the impact and accessibility of findings.<br>• Feedback: Consider converting key tables into visual representations to improve readability and engagement. For example, pie charts or bar graphs could depict the distribution of study locations, types of outcomes measured, or demographic breakdowns. This will make the data more compelling and easier to interpret.	point. We have converted key tables to figures.	studies (Figure 3), and outcomes measured (Figure 4).
5. Feedback on Quality Assessment<br>• Observation: While the study utilizes established tools like the CASP, Oxford level of evidence and JBI checklists for quality assessment, there is insufficient narrative explanation about the scoring process and its implications. The lack of detailed interpretation limits the reader's understanding of how quality influenced the synthesis and conclusions.<br>• Feedback: Recommend including more narrative text to explain:<br>o Scoring Process: Clarify how the quality assessment scores were assigned, who conducted the assessments, and whether assessments were conducted independently or collaboratively.<br>o Implications of Scores: Provide more insight into how the quality scores	Thank you for this comment. We agree and have included more narrative text to cover points raised.	Increased information on quality assessment added in methods:<br><br>“To assess the methodological quality of included studies, we utilized the Critical Appraisal Skills Programme (CASP) tool, and the Joanna Briggs Institute (JBI) critical appraisal checklists.[14,15] Two independent reviewers conducted the quality assessments, with discrepancies resolved through discussion or consultation with a third reviewer. Each study was evaluated using the tool most appropriate for its study design (e.g., CASP for cohort studies research, JBI checklists for case reports). The scores were assigned based on predefined criteria, focusing on factors such as study design, risk of bias, sample representativeness, and methodological rigor. All studies meeting the eligibility criteria were included in the review regardless of quality scores. While lower-quality

impacted the inclusion of studies or the weighting of their findings in the analysis. Or did you include them all and if so why?<br>o Author Commentary: Include a brief discussion of the authors' opinions on the overall quality of the included studies and the implications for the robustness of the review's findings. For example, what does the observed variability in study quality indicate about the current state of research on gender-affirming care?		studies were not excluded, their findings were interpreted with caution, and their methodological limitations were explicitly considered in the synthesis. Studies with higher methodological rigour (e.g., prospective designs, validated outcome measures, and robust statistical analyses) were given greater interpretative weight in the discussion of results. Conversely, studies with high risk of bias or significant methodological concerns (e.g., unclear inclusion criteria, small sample sizes, or lack of validated outcome measures) were acknowledged as contributing to heterogeneity in findings.<br>Increased author commentary added in discussion:<br><br>“The quality assessment revealed considerable variability in study design, methodology, and reporting. While some studies demonstrated strong methodological rigour, a significant proportion had notable limitations, including small sample sizes, retrospective designs, and inconsistent use of validated PROMs. These findings highlight the need for more standardized and high-quality research in gender-affirming care, particularly regarding outcome measurement. The heterogeneity in study quality underscores challenges in synthesizing evidence and reinforces the necessity for future research to adhere to rigorous methodological standards to improve the reliability and generalizability of findings. Despite these limitations, the inclusion of all studies, along with transparent

		quality assessment, provides a comprehensive overview of the existing literature and identifies critical gaps for future research.”
6. Discussion and Policy Implications<br>• Observation: While recommendations are made as to how this could impact policy in Canada and healthcare, the feasibility and implementation pathways are not discussed in depth.<br>• Feedback: Strengthen the discussion by outlining practical steps, resources, and collaborations needed to implement the recommendations. For example:<br>o What institutional, financial, or systemic changes are required?<br>o Which stakeholders should be involved in the process?<br>o Are there existing frameworks or pilot programs that can serve as models? You mentioned the UK’s model, could you expand more?	Thank you for these comments. We have included increased discussion on these important points.	Increased discussion around policy implications included:<br><br>While policies increasingly recognize the importance of gender-affirming care[3,4,9,10], there remains a gap in how outcomes are assessed and integrated into healthcare decision-making. Implementing a national strategy requires systemic, financial, and institutional changes to ensure consistent and meaningful outcome measurement. One key step is the standardization of PROMs in clinical practice. National regulatory bodies, such as the Canadian Medical Association (CMA) and provincial health authorities, should establish guidelines mandating the use of validated PROMs in gender-affirming care. This would ensure consistency across provinces and facilitate comparisons between different healthcare settings, including in key settings such as paediatrics, medical imaging, pharmacy, and endocrinology.[88-91] Additionally, integrating PROMs into electronic health records (EHRs) would enable routine data collection, supporting both clinical decision-making and long-term outcome tracking. Investment in digital infrastructure and interoperability across provincial health systems is essential to facilitate this process. Financial support is crucial for implementation. The

		Canadian Institutes of Health Research (CIHR) and provincial health ministries should allocate dedicated funding for research and pilot programs focused on PROM integration. Financial incentives for clinics and hospitals adopting standardized outcome measures could further encourage widespread implementation, along with transformational healthcare leadership.[92,93] Additionally, insurance providers should explore reimbursement models linked to standardized outcome reporting, ensuring that PROMs play a role in funding decisions for gender-affirming interventions. Stakeholder engagement is essential to ensure that PROMs reflect the diverse needs of transgender and gender-diverse populations. Healthcare providers require training to administer and interpret PROMs effectively, with organizations such as the Canadian Professional Association for Transgender Health (CPATH) and the College of Family Physicians of Canada (CFPC) playing a central role in provider education. Equally important is the involvement of TGD individuals and advocacy groups in co-designing outcome measures to ensure relevance and inclusivity. Establishing a national research consortium on gender-affirming care outcomes, bringing together universities, policymakers, and community organizations, would further support evidence-based policymaking.[94]<br><br>Canada can also learn from the UK's national PROM

		strategy for gender-affirming care, which integrates standardized outcome measures into the National Health Service (NHS) and tracks long-term patient-reported outcomes.[95] A similar federated data-sharing system in Canada could enable outcome comparisons across provinces while protecting patient privacy. The UK model also emphasizes longitudinal data collection, ensuring that patient experiences are captured beyond immediate post-treatment outcomes.[95] Implementing a similar structure in Canada would require coordination between Health Canada, provincial ministries, and research institutions to develop a unified framework. Standardized outcome measurement in gender-affirming care is critical for ensuring equitable, high-quality healthcare. Implementing a national strategy will require systemic reforms, financial investment, and collaboration between healthcare providers, researchers, and policymakers. By building on existing models, particularly the UK's national approach, Canada has an opportunity to lead in evidence-based, patient-centered gender-affirming care. Future research should prioritize pilot studies to assess the feasibility of PROM integration in clinical settings, ensuring that outcome measurement informs both policy and patient care in a meaningful way.
7. Feedback on Word Limit and Depth of Discussion<br>• Observation: At 2,500 words, the document is relatively short for a review paper tackling	Thank you for this comment. We have met as a team and included a significant amount of additional discussion to the paper.	Additional discussion included in line with reviewer suggestions:<br><br>"This review underscores the critical need for standardized PROMs in gender-affirming

such a complex topic as gender-affirming care. This brevity limits the depth of analysis, particularly in the discussion section, where a more critical and analytical approach is needed.<br>• Feedback: Recommend an expansion of the discussion section to provide a deeper, more analytical interpretation of the findings. I would meet as a research team again and think about why are PROM's so important and in particular in this area? What considerations does that have for clinicians, for patients, for activism?		care. In gender-affirming care, where the goal is not just procedural success but improvements in quality of life, social functioning, and mental health, PROMs play an essential role in ensuring that care aligns with patient needs.[8,10] Without standardized measures, it is difficult to assess long-term outcomes, track disparities, or evaluate the effectiveness of interventions, limiting the ability to refine and improve care. For clinicians, integrating PROMs into practice ensures that patient perspectives are systematically incorporated into decision-making, allowing for more individualized and responsive care. Clinicians would benefit from training on how to administer and interpret PROMs effectively, ensuring they are used to guide discussions and treatment planning rather than simply collected as data points.[96] PROMs also hold implications for healthcare systems, as standardized outcome measurement can inform funding decisions, service delivery, and policy development.[95] Clinics and hospitals adopting validated PROMs could be incentivized through funding models that prioritize outcome-based care, ensuring that gender-affirming services are supported at a structural level.<br><br>For patients, the implementation of PROMs has the potential to make care more patient-centered and transparent.[96] When used effectively, PROMs can validate patient experiences and highlight areas where care needs improvement, ensuring that healthcare providers are accountable to the

		communities they serve.[96] However, this requires careful attention to how PROMs are developed, ensuring that they are inclusive, reflective of diverse lived experiences, and accessible across different populations. Beyond clinical applications, PROMs also have broader implications for policy and activism. Standardized outcome measurement can provide the data needed to advocate for improved access to gender-affirming care, demonstrating its impact on mental health and overall well-being. Policymakers can use PROM-driven evidence to justify expanded coverage, reduce wait times, and allocate resources more effectively. Additionally, advocacy groups can leverage PROM data to push for healthcare reforms, ensuring that policies are informed by patient-reported experiences rather than assumptions or incomplete clinical metrics.[94] The UK's national PROM strategy for gender-affirming care offers a useful model, illustrating how systematic outcome measurement can drive evidence-based policy and care improvements.[95]<br><br>Standardizing PROMs in gender-affirming care is not just a methodological concern but a necessary step toward ensuring that care is truly patient-centered, equitable, and evidence-based. Implementation requires collaboration between healthcare providers, researchers, policymakers, and patient communities to ensure that outcome measurement serves its intended purpose: improving care, reducing disparities, and

		reinforcing the legitimacy of gender-affirming healthcare within broader medical and policy frameworks. Future research should focus on piloting PROMs in clinical settings, addressing barriers to their use, and ensuring that they remain responsive to the evolving needs of the transgender and gender-diverse community.<br><br>It is also highly important in PROM implementation to implement a PROM which is not tied to a conflict of interest regarding its developers, or tied to potential licensing fees from for-profit studies, which highlights a broader concern with proprietary PROM development. This is not an isolated issue; proprietary frameworks, which involve licensing agreements and associated fees, are a recurring feature in the development of PROMs across healthcare fields.[97] This is an important issue because the potential collection of licensing fees from for-profit organizations, such as pharmaceutical companies, introduces financial incentives that skew research priorities.[98] The emphasis shifts toward developing tools that generate revenue, particularly for industry-sponsored trials, rather than focusing on the immediate needs of underserved populations, such as transgender and gender diverse people. This model, while commercially viable, risks prioritizing market-driven goals over equity and accessibility. In addition, such models create financial and administrative barriers that limit the accessibility of these tools, particularly in under-

		resourced settings. By requiring licensing agreements, proprietary tools introduce hurdles that discourage their adoption, perpetuating inequities in outcomes measurement. Notable examples of PROMs created using an equity-driven approach, and ready to implement include the Gender Congruence and Life Satisfaction Scale (GCLS)[82], and iTransQoL.[83] These tools are already validated, widely accessible, and capable of capturing key outcomes relevant to TGD populations. These tools also are concise in nature in regard to number of items, which is a key consideration in PROM implementation to implement a PROM which is not burdensome to patients to complete.[96] Using and/or optimizing these tools for TGD-specific contexts represents a resource-conscious and actionable path forward to urgently improve outcome measurement in this area.”

VERSION 2 - REVIEW

Reviewer 2

Name Kearns, Seán

Affiliation University College Dublin, School of Nursing, Midwifery and Health Sciences

Date 11-Feb-2025

COI

Thank you to the authors for your thoughtful consideration of the feedback, best of luck with the publication
